# A systematic review and meta-analysis of unmet needs for healthcare and long-term care among older people

**DOI:** 10.1186/s13561-022-00398-4

**Published:** 2022-12-09

**Authors:** Md. Mizanur Rahman, Megumi Rosenberg, Gabriela Flores, Nadia Parsell, Shamima Akter, Md Ashraful Alam, Md. Mahfuzur Rahman, Tessa Edejer

**Affiliations:** 1 Hitotsubashi Institute for Advanced Study, Hitotsubashi University, Tokyo, Japan; 2 Centre for Health Development, World Health Organization, Kobe, Japan; 3 Department of Health Systems Governance and Financing, World Health Organization, Geneva, Switzerland; 4 Department of Computational Diagnostic Radiology and Preventive Medicine, The University of Tokyo Hospital, Tokyo, Japan; 5 Global Public Health Research Foundation, Dhaka, Bangladesh

**Keywords:** Unmet need, Long-term care, Barrier dimension, Older population, Systematic review, meta-analysis, Md. Mizanur Rahman and Megumi Rosenberg are jointly first author.

## Abstract

**Background:**

The absolute number of older individuals needing medical care and long-term care (LTC) is increasing globally due to the growing ageing population. However, it is uncertain who and what proportion of the population has access to care. Therefore, a systematic review and meta-analysis of the prevalence and reasons for unmet needs for healthcare and long-term care among older people, 65 years old and above, across countries was conducted.

**Methods:**

An information specialist performed a comprehensive search of four major databases (PubMed, EMBASE, Web of Science, and CINAHL) from inception to June 2020 without restrictions on language and date. We did random-effects meta-analysis to obtain pooled prevalence. We stratified the meta-analysis by reasons for unmet need categorized by barrier dimension (availability, accessibility, affordability, and acceptability), survey year, geographic location, and socio-demographic characteristics of the older individual.

**Results:**

After screening 3912 articles, we included 101 studies published between 1996 and 2020. Of the 101 studies, 87 studies reported unmet healthcare needs and 14 studies reported unmet LTC needs. Overall, 10.4% (95% CI, 7.3–13.9) of the older population had unmet needs for healthcare. The common reasons for unmet healthcare needs were cost of treatment, lack of health facilities, lack of/conflicting time, health problem not viewed as serious, and mistrust/fear of provider. A significant variation in pooled prevalence of unmet healthcare needs due to cost was found by gender (male [10.9, 95% CI, 8.9–13.1] vs female [14.4, 95% CI, 11.8–17.3]), educational level (primary or less [13.3, 95% CI, 9.6–17.6] vs higher [7.5, 95% CI, 5.9–9.3]), self-reported health (poor [23.2, 95% CI, 18.8–27.8] vs good [4.4, 95% CI, 3.4–5.5]), insurance status (insured [9.0, 95% CI, 7.5–10.6] vs uninsured [27.7, 95% CI, 24.0–31.5]), and economic status of population (poorest [28.2, 95% CI, 14.1–44.9] vs richest [7.1, 95% CI, 3.8–11.3]). One in four (25.1, 95% CI, 17.1–34.2) older people had unmet needs in LTC. Rural residents had a higher prevalence of unmet needs in LTC compared to their urban counterparts.

**Conclusion:**

With the population ageing globally, it is necessary to improve access to health care and LTC for older people. Ensuring affordability of health services, reducing geographical barriers, and improving acceptability, will be critical in reducing unmet need. Unmet needs for healthcare were concentrated in population with no education, poor economic group, outpatient health facility user, and uninsured group. With education and economic-based inequalities at the forefront, all countries should focus on improving access to health services by reducing the burden related to healthcare costs.

**Supplementary Information:**

The online version contains supplementary material available at 10.1186/s13561-022-00398-4.

## Background

Globally, the older population is rapidly growing, with the number of adults over 60 projected to double and that over 80 to triple from 2017 to 2050 [1]. In turn, the absolute number of older adults needing quality care will also increase [2–4]. Longitudinal studies tracking healthy ageing from 26 countries found that 71.4% of participants demonstrated stable and healthy ageing over a span of 10 years [5]. However, 25.2% of participants had stable but low health and 3.4% of participants experienced rapid deterioration in their health over time. As such, it is necessary to ensure all older people have access to quality and affordable social services, including healthcare and long-term care (LTC).

One method of measuring access to care is by estimating the proportion of the population that has unmet needs. An individual is categorized as having unmet needs if they are unable to access quality care when needed [6]. Unmet needs can arise for various reasons, including barriers related to the availability, affordability, accessibility, and acceptability of services [7]. Among older people, unmet needs for health services have been associated with adverse outcomes such as increased mortality [8] and depression [9]. While healthcare refers to medical services needed to promote, restore, or maintain health, LTC refers to assistance with activities of daily living (ADL), such as walking, eating, and bathing, and instrumental activities of daily living (IADL), such as cleaning and cooking [10]. LTC is necessary for the well-being and safety of older people with functional and/or cognitive impairments [11]. Unmet needs for LTC have been associated with increased risk of hospital admission [12], hospital readmission [13], emergency department admission for falls and injuries [14], and mortality [15]. Therefore, it is necessary to identify who is not accessing care and uncover the reasons why so that policies and interventions can be tailored to protect the health of older persons at risk.

Although there are many original studies that have examined unmet needs [16–33], to the best of our knowledge, there has been no systematic review and meta-analysis of unmet needs for healthcare or LTC among older people. Since there is great variability in prevalence and reasons for unmet healthcare needs across studies [15, 33–41], our main objective is to provide a pooled estimate of unmet needs for healthcare among older people across countries and socio-demographic groups, as well as to identify the leading reasons for those unmet needs. In addition, our secondary objective is to estimate the proportion of unmet needs for LTC among the older population.

## Methods

### Literature search

We performed a systematic review and meta-analysis of observational studies assessing unmet healthcare needs. This study addressed several research questions such as What is the prevalence of unmet need for health care? What are its main drivers? How does it vary by age group, poverty status, gender and education level, geographic location (e.g. rural/urban), insurance scheme and other socio-economic status? Supply side factors (e.g. insurance scheme, availability of long-term care insurance, service location) and broader macroeconomic factors (e.g. country income group)? This study followed Preferred Reporting Items for Systematic Reviews and Meta-Analyses (PRISMA) guidelines for reporting the manuscript [42]. An information specialist did a comprehensive literature search on June 24, 2020, for relevant articles published from inception to June 24, 2020, in the following databases: PubMed, Embase, Web of Science, and CINAHL. No date or language restrictions were applied during the database search. To identify the relevant papers, we combined with “AND” operators of three major topics: (forgone health care OR unmet needs) AND (barrier for healthcare OR long-term care) AND quantitative survey. Further searches for eligible studies were conducted by reviewing references within identified papers. The details of the search strategy are presented in the Appendix (Table S1-S4).

### Inclusion and exclusion criteria

The inclusion criteria were an original article, use of household/community/facility level survey data, use of quantitative analysis, and reporting on outcomes on either forgone care or unmet needs related to healthcare or LTC. Countries at all income levels and all World Health Organization (WHO) regions were included in this study. We excluded qualitative studies, letters, case series, reviews, commentaries, and editorials. Studies based on specific diseases or patient groups were also excluded. Following the study inclusion and exclusion criteria, two independent reviewers first screened the title and abstract (AS, RMM, AM, and RM), and then selected full texts. Any discrepancy among the reviewers during the two stages were resolved through discussions with RM, FG, and ET.

### Quality assessment of methodology of the studies

The New-Castle Ottawa Scale (NOS) Tool was used to assess the study quality for observational studies. We classified the studies as of high, moderate, and low qualities, based on their total scores as follows: high if they scored ≥6, moderate if they scored 4–5, and low if they scored 0–3. Two reviewers independently assessed the study quality, which were then cross-checked by two other authors. Any discrepancies found were resolved through discussion.

### Data analysis

A pilot-tested data extraction form was used to collect information from the included articles. Extracted data included the first author’s last name, study country, publication year, survey year, study design, sample size, age range, outcome variables, recall period of outcome variable, barrier framework, explanatory framework, and reasons for unmet needs. We recorded prevalence and event of unmet needs at the overall level and by strata, such as by age, gender, education, occupation, marital status, economic group, migrant status, type of health facilities used, insurance status, geographic location, and type of diseases. Furthermore, we compiled reasons for unmet needs when available. The data extraction form and detailed information of the extracted variables are presented in the Appendix (eMethod1).

The primary outcome of interest was unmet healthcare needs and the secondary outcome was unmet needs in LTC. We generally followed the definitions for unmet needs for healthcare or LTC used in the original studies [17, 22, 24, 43–45]. In addition, in the present study, we included foregone care, not receiving necessary care, delaying needed medical, dental, or pharmacy care in our definition of unmet healthcare needs. These terms were extracted from key papers and used to corroborate the search strategy used in identifying the original papers. Most studies referring to forgone healthcare measured it by asking questions such as, “Was there a time in the past year you needed a type of (health) care but did not get it? [19, 46–51] Likewise, studies referring to unmet needs for healthcare measured this by asking questions like, “During the past 12 months, was there ever a time when you felt that you needed health care but didn’t receive it?” [30, 33, 38, 45, 52] Although the key word (unmet needs and forgone care) is different, the question actually collects the same information. Therefore, forgone care was included in our definition of unmet healthcare needs. For similar reasons, non-receipt of needed care and delayed care were also included as unmet healthcare needs in our study. The reasons for unmet healthcare needs were derived from survey questions such as, “Thinking of the most recent time (that you didn’t get care when you needed it), why didn’t you get care?” [33, 38, 52]

With regard to defining unmet needs for LTC, previous studies tend to define unmet needs for LTC among older people based on when a person has needs for assistance with ADL or IADL, but the assistance is unavailable, insufficient, or had to wait [14, 53]. The simplest way to define unmet need is to define the population with LTC needs and assess whether they received assistance. We followed the definition of unmet needs for LTC used in the original studies. Further information about how unmet needs in healthcare and LTC were operationalized in this study are presented in the Appendix (eMethod2).

We used prevalence estimates (i.e. proportion of the population with the outcome of interest) for the meta-analysis. When necessary, we calculated prevalence using the original study data provided in the publications. Fixed-effect or random effects meta-analysis was performed depending on the degree of heterogeneity. We used *I*^2^ statistic to assess the level of statistical heterogeneity between the included studies. Based on previous studies, *I*^2^ of < 50 indicated low heterogeneity, between 50 and 75% indicated moderate heterogeneity, and greater than 75% indicated high heterogeneity. We summarized the study-specific estimates using a random-effects model to obtain a pooled prevalence of unmet needs [54]. Furthermore, we summarized unmet needs for the following subgroups: the older population (age 65 and above) stratified by reason for unmet needs/barrier dimension, gender (male or female), level of education (primary or less, secondary or college, or higher), self-reported health status (poor/fair, good/average, or very good/excellent), type of illness (NCDs/chronic condition or depression symptoms), insurance enrollment status (insured or uninsured), level of income or socioeconomic status (by quintile, i.e., poorest, poorer, average, rich, or richest), place of residence (urban or rural), and survey year (≤2000, 2001–2010, or 2011–2019). All analyses were performed using Stata version 16.1/MP (StataCorp, College Station, TX, USA).

## Results

### Study characteristics

**Fig. 1:**
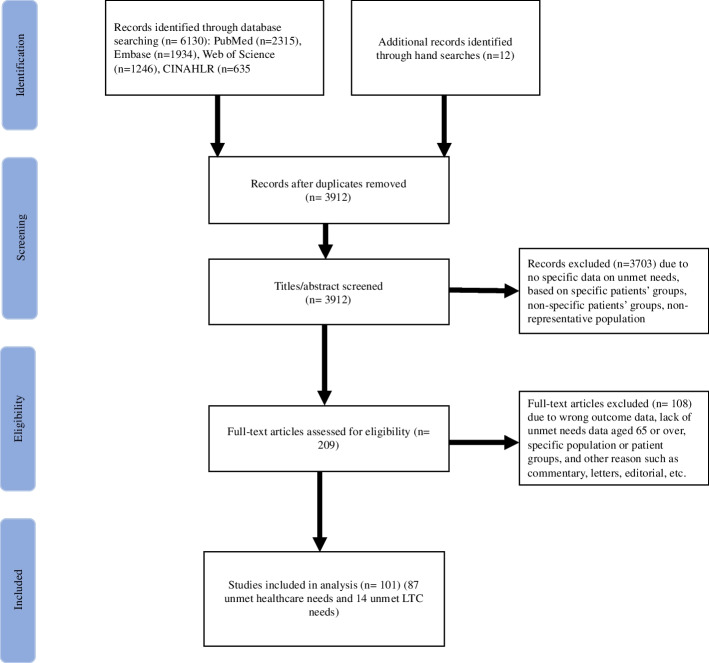
PRISMA flowchart for study selection. LTC, long-term care

The electronic databases identified 6130 articles. The grey literature search and review of relevant references identified 12 more articles. After removal of duplicates, 3912 articles were eligible for title and abstract screening, resulting in 209 articles that were extracted for full text review. After reviewing the full text, 101 articles were included in the systematic review and meta-analysis (Fig. 1). Of these, 87 studies reported unmet needs for healthcare among the older population, 65 years and older, as outcome variables and 14 studies focused only on unmet LTC needs. The included studies were conducted as early as 1996 and up to 2020. More than 90% of the studies were conducted in the United States of America (USA) and European countries and very few in the Asian and African regions. The study characteristics of the included papers are summarized in the Appendix (Table S5-S6). Most of the included studies were of high quality (Table S7-S10).

### Prevalence estimate across different types of care

Table 1 shows the summary estimates of unmet needs for healthcare generally and, in cases where it was specified in the study, by type of care. In the summary analysis, the numerator was the total number of older individuals that had unmet needs and the denominator was the total older population. On average, 10.0% of the included population had unmet healthcare needs due to any reason. Prevalence of unmet need differed by type of care in the older population: unmet need was highest for prescription/medications (15.0%, 4 studies) and the lowest for checkups/examinations (7.7%, 3 studies).

**Table 1:** Summary estimates of unmet needs for healthcare among older people, 65 years and older (*N* = 87 studies)

Unmet need for healthcare	No. of studies	Frequency of unmet needs population	Pooled prevalence (95% CI)
**Overall (Type of care not specified)**	**73**	5,494,058	10.0 (9.2–10.9)
**By type of care (Only when specified)**			
Unmet needs for medical care	4	1858	10.4 (7.3–13.9)
Unmet needs for dental care	6	2094	8.8 (5.6–12.7)
Unmet needs for checkup/examination	3	471	7.7 (4.8–11.2)
Unmet needs for prescription/medications	4	507	15.0 (10.5–20.0)

*Note*: In this summary estimate, numerator was total number of older population who reported unmet needs for healthcare and denominator was total number of older population

### Reasons for unmet healthcare needs

**Fig. 2:**
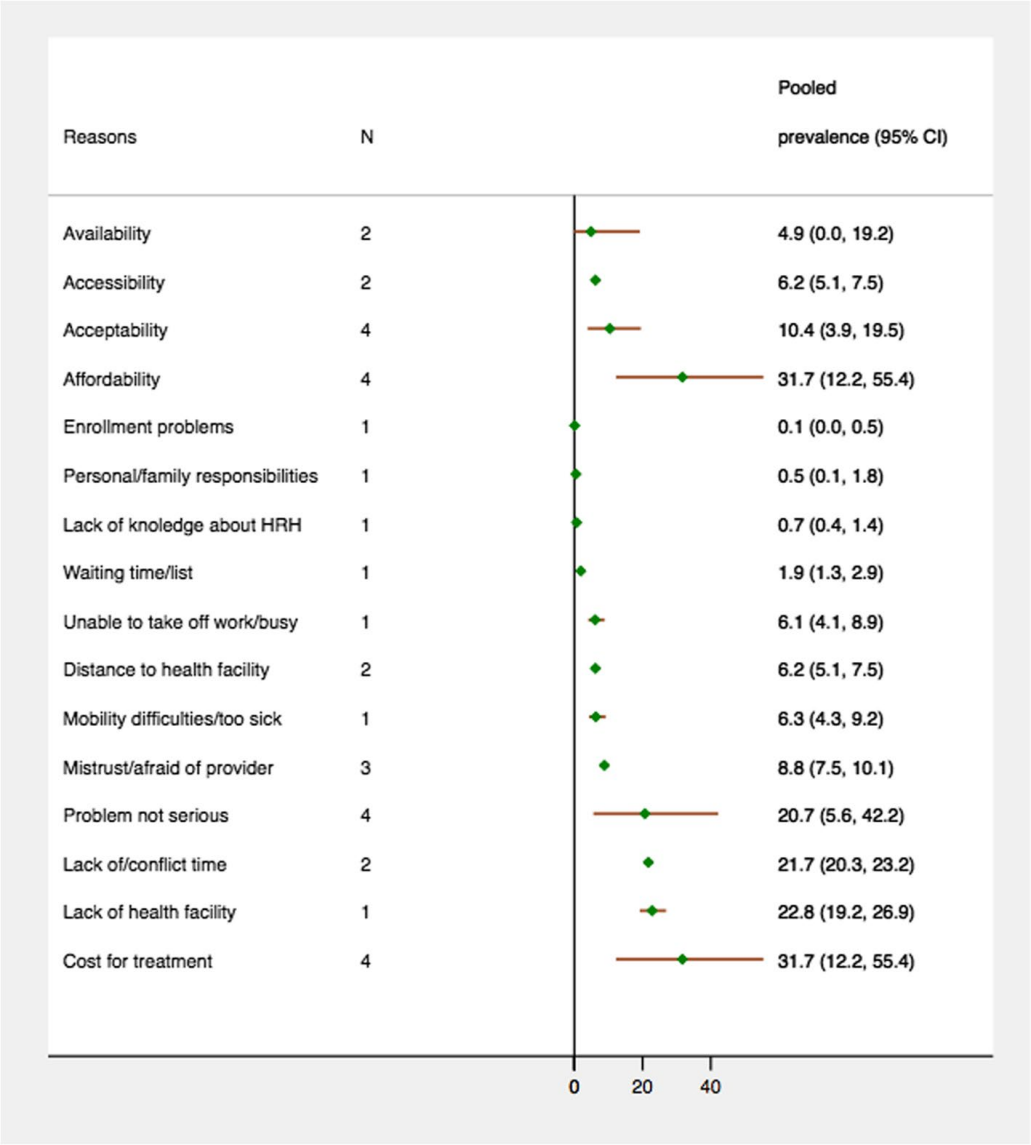
Reason specific prevalence of unmet needs for healthcare among older people

We classified reasons for unmet needs for healthcare into four barrier dimensions: availability, accessibility, affordability, and acceptability. In this sub-group analysis, the numerator was the total number of individuals that reported a reason for unmet healthcare needs related to a specific barrier dimension and the denominator was the total number of individuals that reported unmet healthcare needs. Figure 2 presents the prevalence of unmet healthcare needs associated with each barrier dimension and the detailed reasons for unmet healthcare needs among the older population. Among the older people that reported unmet healthcare needs, the leading barrier dimension was for problems of affordability (31.7%, 4 studies), followed by acceptability (10.4%, 4 studies), accessibility (6.2%, 2 studies) and availability (4.9%, 2 studies). In case of detailed reasons, the most commonly reported reasons for unmet healthcare need were cost of treatment (31.7%, 4 studies), lack of health facilities (22.8%, 1 study), lack of/conflicting time (21.7%, 2 studies), health problem viewed as not serious (20.7%, 4 studies), mistrust/fear of providers (8.8%, 3 studies), mobility difficulties/too sick (6.3%, 1 study), distance to health facility (6.2%, 2 studies), and unable to take off work/busy (6.1%, 1 study).

### Subgroup analysis by socio-demographic characteristics and survey year

Prevalence of unmet healthcare needs among older people differed significantly by socio-demographic characteristics (Table 2). Focusing on unmet healthcare needs due to cost-related reasons, which was the most common, there were significant variations in prevalence by gender (male [10.9%] vs female [14.4%]), educational level (primary or less [13.3%] vs higher [7.5%]), self-reported health (poor [23.2%] vs good [4.4%]), insurance status (insured [9.0%] vs uninsured [27.7%]), and economic status of population (poorest [28.2%] vs richest [7.1%]). Rates of unmet needs for healthcare among older people significantly increased from 4.3% in studies conducted during 2001–2010 to 10.8% in studies conducted during 2011–2019. Almost similar pattern was found in case unmet healthcare needs due to any reasons. The details country-specific prevalence of unmet healthcare needs among older people is presented in the supplemental Appendix (Fig. S1-S2).

**Table 2:** Unmet need for healthcare among the older population by selected variables and by reasons for the unmet need for healthcare

Characteristics	Due to any reason		Due to cost/economic reasons	
	No. of studies	Prevalence (95% CI)	No. of studies	Prevalence (95% CI)
**Gender**		(*p* = 0.8)		**(*p*** **< 0.01)**
Male	31	12.5 (7.1–19.2)	20	10.9 (8.9–13.1)
Female	31	13.6 (11.0–16.4)	22	14.4 (11.8–17.3)
**Educational level**		(*P* = 0.3)		**(*p*** **< 0.01)**
Primary or less	14	11.4 (9.0–14.1)	13	13.3 (9.6–17.6)
Secondary or college	18	12.3 (10.0–14.0)	11	12.2 (9.3–15.3)
Higher	12	14.5 (11.6–17.8)	13	7.5 (5.9–9.3)
**Self-reported health**		**(*p*** **< 0.01)**		**(*p*** **< 0.01)**
Poor/fair	16	20.2 (17.0–23.6)	9	23.2 (18.8–27.8)
Good/average	14	17.3 (10.9–24.8)	6	4.4 (3.4–5.5)
Very good/excellent	14	11.6 (9.7–13.7)	5	13.8 (8.7–19.8)
**Type of illness**		(*p* = 0.7)		(*p* = 0.8)
Have NCD/chronic condition	10	7.1 (5.8–8.5)	10	12.8 (7.9–18.8)
Have depression symptoms	6	6.2 (2.0–12.4)	7	13.6 (13.1–14.1)
**Insurance status**		(*P* = 0.3)		**(*p*** **< 0.01)**
Insured^a^	14	14.4 (9.5–20.0)	9	9.0 (7.5–10.6)
None	13	18.0 (14.8–21.4)	10	27.7 (24.0–31.5)
**Income/SES group**		**(*p*** **< 0.01)**		**(*p*** **< 0.01)**
Q1 (Poorest)	18	23.6 (19.3–28.3)	8	28.2 (14.1–44.9)
Q2	17	13.9 (10.4–17.9)	7	15.3 (8.6–23.4)
Q3	17	11.2 (6.6–16.9)	7	10.1 (5.6–15.8)
Q4	14	9.2 (6.8–11.8)	6	9.7 (4.6–16.4)
Q5 (Richest)	11	6.5 (5.0–8.1)	7	7.1 (3.8–11.3)
**Residence**		(*p* = 0.9)		(*p* = 0.3)
Urban	13	14.4 (11.5–17.6)	5	12.8 (9.0–17.2)
Rural	15	14.7 (11.2–18.6)	5	16.2 (11.8–21.2)
**Survey year**		**(*p*** **< 0.01)**		**(*p*** **< 0.01)**
≤ 2000	8	12.4 (7.8–17.8)	3	7.4 (3.2–13.2)
2001–2010	34	15.5 (12.1–19.2)	14	4.3 (3.1–5.8)
2011–2019	28	10.1 (8.3–11.9)	19	10.8 (9.9–11.8)

^a^Type of insurance included public/private/other types

*Note*: In this stratified analysis, numerator was total number of older population who reported unmet needs for healthcare and denominator was total number of older population in each stratifier

### Unmet LTC needs

**Fig. 3:**
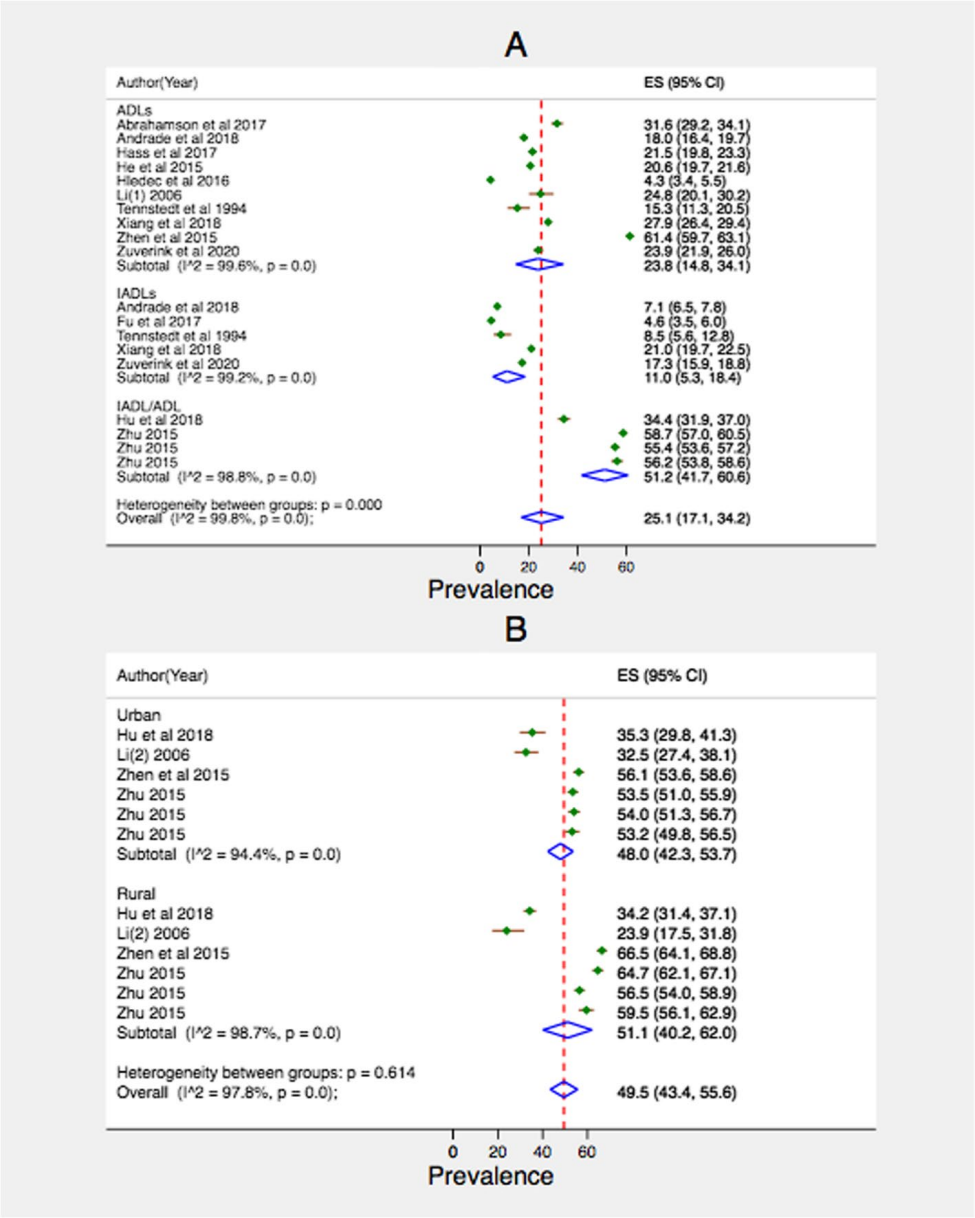
Pooled prevalence of unmet long-term care needs among the older population. A, by health condition and overall; B, by place of residence

Figure 3 presents the pooled prevalence of unmet needs for LTC among older people. On average, 25.1% of older people had unmet needs for LTC (13 studies). The prevalence of unmet need for care related to ADLs (23.8%) was higher than that related to IADLs (11.0%). Rural residents had a higher prevalence of unmet needs in LTC (51.1%, 4 studies) compared to their urban counterparts (48.0%, 4 studies).

## Discussion

The main objective of this systematic review and meta-analysis was to estimate the prevalence of unmet needs for healthcare among the older population across socio-demographic groups and to understand the reasons for unmet needs. Furthermore, given the importance of LTC for the well-being of older individuals with physical and/or cognitive limitations and their family caregivers, we additionally estimated unmet needs for LTC.

On average, 10.0% of the included older population had unmet healthcare needs and this prevalence differed by type of care: unmet need was highest for prescription/medications (15.0%, 4 studies) and the lowest for checkups/examinations (7.7%, 3 studies). The study findings indicated that affordability-related problems, such as cost of treatment, were the most frequently reported reasons for unmet needs, with other major barriers including lack of health facilities, mistrust/fear of provider, and lack of time/conflicting time schedules. Moreover, a fifth of the total included population had unmet needs because they did not perceive their health concerns to be serious enough to warrant medical care.

Those who need the most care tend to have the least access since utilization of services is heavily influenced by socioeconomic factors [55]. Our results suggest that older people with poorer self-reported health, those with primary or less education, and those in the poorest quintile have higher unmet needs than their respective counterparts. Most often, disadvantaged groups have less access to healthcare and are more likely to be exposed to behavioral risk factors (i.e., tobacco, unhealthy foods, and alcohol) that result in poorer health outcomes [55, 56]. Additionally, our findings revealed that women report more unmet needs than men. Although women live longer than men, women spend more years living with disability and are more likely to be in poverty and report difficulties affording care [57]. Factors such as lower wages and less years in paid employment (due to role as primary caregiver) may result in less financial resources to pay for healthcare [58, 59].

In addition to increased need for medical care, most older adults will eventually need assistance for LTC as their physical and/or cognitive abilities start to decline. Overall, we found that a quarter of the older population had unmet LTC needs, with higher unmet needs for ADL than for IDL. Having unmet need for LTC, especially for ADL, can place older individuals in danger of injuries, falls, and death [14, 22]. Furthermore, the subgroup analysis found unmet needs for LTC to be higher in rural areas. While most LTC is provided by families, formal LTC may be limited in rural areas due to limited supply of skilled workers, community-based care, and institutional care services [60]. Globally, the need for formal (paid) LTC and government assistance to cover costs are rising [55]. Countries such as Sweden, the Netherlands, and Japan have been able to fund and provide comprehensive LTC through financing mechanisms such as general taxes and social insurance schemes (compulsory payments through payroll/income) [61]. As the demand for LTC is expected to sharply increase due to global population ageing, important lessons on how to finance and structure LTC can be drawn from such countries that have been adapting their health and social care systems to the changing needs of an aging population.

### Strength and limitations

The use of comprehensive search techniques and validated systematic review methods, following the Preferred Reporting Items for Systematic Reviews and Meta-Analyses (PRISMA) guideline [42], strengthens our conclusions. We investigated the prevalence of unmet needs for healthcare in the general population and specifically among older people across countries, by socio-demographic groups, and over time. Furthermore, we used the appropriate statistical techniques to estimate pooled prevalence of unmet needs for healthcare and LTC and identified the leading reasons for unmet needs among the older population. We gave visibility to a critical dimension of unaffordability (unmet needs for financial reasons) but it is beyond the scope of this paper to study financial hardship arising from out-of-pocket payments (the other dimension of unaffordability) [62–65]. Despite these strengths, there are several limitations to this study. First, most of our included studies were from countries in Europe and the USA while only a few were from countries in Asia and Africa. Therefore, our findings are not inclusive of all countries in the world. Second, we were not able to perform detailed subgroup analysis for unmet needs for LTC due to lack of available data. Third, we found severe heterogeneity of prevalence in the included studies. To explain this heterogeneity, we conducted stratified analyses by survey year, sample size, and other participant level characteristics.

## Conclusion

Although this study found a high level of heterogeneity in the prevalence of unmet needs among the older population across studies, our findings suggest that unmet needs for healthcare among older people are mainly due to cost of treatment, lack of health facilities, lack of/conflicting time, health problem not viewed as serious, and mistrust/fear of the provider. Prevalence of unmet needs are more prevalent among older people in disadvantaged population groups. Financial protection policies need to be strengthened in the studied countries to remove financial barriers to care and ensure equity in service coverage. This should consider older people’s needs for chronic healthcare and LTC in the context of global population ageing. Based on the findings of this study, the following policy recommendations are as follows:

- Increase government expenditure on health, invest in compulsory social health insurance programs, and subsidize premiums for the disadvantaged population including poor, uninsured, and unemployed population.
- Invest in affordable and reliable transportation to health facilities for rural residents.

## Supplementary Information

**Additional file 1: Table S1.** PubMed search results (June 24, 2020). **Table S2.** EMBASE search results (June 24, 2020). **Table S3.** Web of Science search results (June 24, 2020). **Table S4.** CINAHL search results (June 24, 2020). **Table S5.** Background characteristics of the study (*N*=87). **Table S6.** Background characteristics of unmet long-term care needs study (*N*=14). **Table S7.** Quality assessment of the cross-sectional studies to unmet needs. **Table S8.** Quality assessment of the Cohort studies related to unmet needs. **Table S9.** Quality assessment of the Cross-sectional studies related to long-term care. **Table S10.** Quality assessment of the Cohort-sectional studies related to long-term care. **Figure S1.** Country-specific prevalence for forgone healthcare due to cost-related reasons among older people, 65 years and above. **Figure S2.** Unmet needs for healthcare among older people due to any reason by country.

## Data Availability

The is not publicly available. The corresponding author is fully responsible for data gathering.
