# Supplementary material for: A systematic review and meta-analysis of unmet needs for healthcare and long-term care among older people
Source: Health Econ Rev. 2022 Dec 9;12:60. doi: 10.1186/s13561-022-00398-4 (PMC9733388; doi:10.1186/s13561-022-00398-4)
Supplement: Supplementary file 1 — Additional file 1: Table S1. PubMed search results (June 24, 2020). Table S2. EMBASE search results (June 24, 2020). Table S3. Web of Science search results (June 24, 2020). Table S4. CINAHL search results (June 24, 2020). Table S5. Background characteristics of the study (N=87). Table S6. Background characteristics of unmet long-term care needs study (N=14). Table S7. Quality assessment of the cross-sectional studies to unmet needs. Table S8. Quality assessment of the Cohort studies related to unmet needs. Table S9. Quality assessment of the Cross-sectional studies related to long-term care. Table S10. Quality assessment of the Cohort-sectional studies related to long-term care. Figure S1. Country-specific prevalence for forgone healthcare due to cost-related reasons among older people, 65 years and above. Figure S2. Unmet needs for healthcare among older people due to any reason by country. [file 13561_2022_398_MOESM1_ESM.docx]

**Supplemental appendix**

**A systematic review and meta-analysis of unmet needs for healthcare and long-term care among the older population**

**Table S1: PubMed search results (June 24, 2020)**

| No. | Query | Results |
| --- | --- | --- |
| #1 | (forgone [tw] OR foregone [tw] OR forgo [tw] OR foregone care [tw] OR forgone care [tw] OR “foregone health service” [tw] OR foregone health care [tw] OR foregone healthcare [tw] OR unmet needs [tw] OR unmet need [tw]) | **14,863** |
| #2 | (barrier [tw] OR barriers [tw] OR obstacle [tw] OR utilization [tw] OR "health services"[MeSH] OR "health services"[tw] OR “access to service” [tw] “access to care” [tw] OR healthcare [MeSH] OR health care [tw] OR healthcare [tw] OR "delivery of health care" [MeSH] OR access to treatment [MeSH] OR “access to treatment” [tw] OR "Health Services Accessibility" [MeSH] OR "health services accessibility" [tw] OR access to medicine [tw] OR (access [tw] AND ("medicine"[MeSH] OR "medicine"[tw])) OR “access to product” [tw] OR “access to surgery” [tw] OR ("long-term care"[MeSH] OR "long-term care"[tw] OR " long term care" [tw] OR "LTC" [tw])) | **1,630,795** |
| #3 | (household survey [tw] OR “Survey of Health, Ageing and Retirement” OR SHARE OR “global ageing and adult health” OR SAGE OR “Health and Retirement Study” OR “Retirement Longitudinal Study” OR “Demographic and Health Surveys” OR DHS OR “Multiple Indicator and Cluster Survey” OR MICS OR “Medical Expenditure Panel Survey” OR Survey [MeSH]) | **1,416,169** |
|  | **#1 AND #2 AND #3** | **2,315** |

**Table S2: EMBASE search results (June 24, 2020)**

| No. | Query | Results |
| --- | --- | --- |
| #4 | **#1 AND #2 AND #3** | **1934** |
| #3 | 'household survey':ti,ab,kw OR ('survey of health, ageing':ti,ab,kw AND retirement:ti,ab,kw) OR share:ti,ab,kw OR ('global ageing':ti,ab,kw AND 'adult health':ti,ab,kw) OR sage:ti,ab,kw OR (health:ti,ab,kw AND 'retirement study':ti,ab,kw) OR 'retirement longitudinal study':ti,ab,kw OR (demographic:ti,ab,kw AND 'health surveys':ti,ab,kw) OR dhs:ti,ab,kw OR ('multiple indicator':ti,ab,kw AND 'cluster survey':ti,ab,kw) OR mics:ti,ab,kw OR 'medical expenditure panel survey':ti,ab,kw OR survey:ti,ab,kw | 870610 |
| #2 | barrier:ti,ab,kw OR barriers:ti,ab,kw OR obstacle:ti,ab,kw OR utilization:ti,ab,kw OR 'health service':ti,ab,kw OR 'health services':ti,ab,kw OR 'access to service':ti,ab,kw OR 'access to care':ti,ab,kw OR healthcare:ti,ab,kw OR 'health care':ti,ab,kw OR 'delivery of health care':ti,ab,kw OR 'access to treatment':ti,ab,kw OR 'health services accessibility':ti,ab,kw OR 'access to medicine':ti,ab,kw OR (access:ti,ab,kw AND 'medicine':ti,ab,kw) OR 'access to product':ti,ab,kw OR 'access to surgery':ti,ab,kw OR ‘long-term care’:ti,ab,kw | 1416026 |
| #1 | forgone:ti,ab,kw OR foregone:ti,ab,kw OR forego:ti,ab,kw OR 'foregone care':ti,ab,kw OR 'forgone care':ti,ab,kw OR 'foregone health service':ti,ab,kw OR 'foregone health care':ti,ab,kw OR 'foregone healthcare':ti,ab,kw OR 'unmet needs':ti,ab,kw OR 'unmet need':ti,ab,kw | 23808 |

**Table S3: Web of Science search results (June 24, 2020)**

| No | Query | Results |
| --- | --- | --- |
| #1 | TI = (“forgone” OR “forego” OR “foregone care” OR “foregone health service” OR “foregone health care” OR “foregone healthcare” OR “unmet needs” OR “unmet need”) OR TS = (“forgone” OR “forego” OR “foregone care” OR “foregone health service” OR “foregone health care” OR “foregone healthcare” OR “unmet needs” OR “unmet need”) | **16,205** |
| #2 | TI = (“barrier” OR “barriers” OR “obstacle” OR “utilization” OR “health services” OR “access to service” OR “access to care” OR “access to treatment” OR “access to medicine” OR “access to product” OR “access to surgery” OR “health care” OR “delivery of health care” OR “health services accessibility” OR “long-term care” OR “LTC“) OR TS = (“barrier” OR “barriers” OR “obstacle” OR “utilization” OR “access to service” OR “access to care” OR “access to treatment” OR “access to medicine” OR “access to product” OR “access to surgery” OR “health care” OR “delivery of health care” OR “health services accessibility” OR “long-term care” OR “LTC“) | **1,429,925** |
| #3 | TI = (“household survey” OR “Survey of Health, Ageing and Retirement” OR “SHARE” OR “global ageing and adult health” OR “SAGE” OR “Health and Retirement Study” OR “Retirement Longitudinal Study” OR “Demographic and Health Surveys” OR “DHS” OR “Multiple Indicator and Cluster Survey” OR “MICS” OR “Medical Expenditure Panel Survey” OR “Survey”) OR TS = (“household survey” OR “Survey of Health, Ageing and Retirement” OR “SHARE” OR “global ageing and adult health” OR “SAGE” OR “Health and Retirement Study” OR “Retirement Longitudinal Study” OR “Demographic and Health Surveys” OR “DHS” OR “Multiple Indicator and Cluster Survey” OR “MICS” OR “Medical Expenditure Panel Survey” OR “Survey”) | **1,267,050** |
|  | **#1 AND #2 AND #3** | **1,246** |

**Table S4: CINAHL search results (June 24, 2020)**

| Search | Query | Items found |
| --- | --- | --- |
| S1 | TI (“forgone” OR “forego” OR “foregone care” OR “foregone health service” OR “foregone health care” OR “foregone healthcare” OR “unmet needs” OR “unmet need”) OR AB (“forgone” OR “forego” OR “foregone care” OR “foregone health service” OR “foregone health care” OR “foregone healthcare” OR “unmet needs” OR “unmet need”) | 6,820 |
| S2 | TI (“barrier” OR “barriers” OR “obstacle” OR “utilization” OR “access to service” OR “access to care” OR “access to treatment” OR “access to medicine” OR “access to product” OR “access to surgery” OR “health care” OR “delivery of health care” OR “health services accessibility” OR “long-term care” OR “LTC“) OR AB (“barrier” OR “barriers” OR “obstacle” OR “utilization” OR “access to service” OR “access to care” OR “access to treatment” OR “access to medicine” OR “access to product” OR “access to surgery” OR “health care” OR “delivery of health care” OR “health services accessibility” OR “long-term care” OR “LTC“) | 344,809 |
| S3 | TI (“household survey” OR “Survey of Health, Ageing and Retirement” OR “SHARE” OR “global ageing and adult health” OR “SAGE” OR “Health and Retirement Study” OR “Retirement Longitudinal Study” OR “Demographic and Health Surveys” OR “DHS” OR “Multiple Indicator and Cluster Survey” OR “MICS” OR “Medical Expenditure Panel Survey” OR “Survey”) OR AB (“household survey” OR “Survey of Health, Ageing and Retirement” OR “SHARE” OR “global ageing and adult health” OR “SAGE” OR “Health and Retirement Study” OR “Retirement Longitudinal Study” OR “Demographic and Health Surveys” OR “DHS” OR “Multiple Indicator and Cluster Survey” OR “MICS” OR “Medical Expenditure Panel Survey” OR “Survey”) | 274,207 |
|  | **#1 AND #2 AND #3** | 635 |

**eMethod1: Data Extraction Form**

**Section 1: Background information**

| Author (Last name et al) |  |
| --- | --- |
| Title |  |
| Year of publication |  |
| Country |  |
| Place  *(Urban/Rural/others)* |  |
| Region |  |
| Study subjects  *(Female/children)* |  |
| Age of the participants (Years) |  |
| Mean or median age of participants |  |
| Study design  *(Cross-section, cohort, case-control, panel study)* |  |
| Survey start |  |
| Survey end |  |
| Sample size |  |
| No. of site/facilities/etc. |  |
| Sampling methods |  |
| Response rate |  |
| Please specify covariates adjusted in analysis |  |

**Section 2: Evidence gap**

| Questions | Response |
| --- | --- |
| **Q2.1 Type of quantitative research:** Descriptive, Correlational, Causal-Comparative/Quasi-Experimental/ Experimental Research/other  *[Please report response right side]* |  |
| **Q2.2 Type of survey: H***ealth survey & HECs stands for standard measurement survey (LSMS), socio-economic survey, household income and expenditure survey, household budget survey, survey of income and living conditions*  *[Please report response right side]* |  |
| Q2.3 B**arriers framework identified:** Yes/No  *[please check the paper if a distinction between barriers framework and explanatory framework below is relevant]* |  |
| **Q2.4 Types of barries framework followed :**  **Barrier framework :** Tanahashi, 1978; Peters et al, 2008; Jacobs et al, 2011; Levesque et al, 2013 ; other.  *[Please report framework on the right side-response section]* |  |
| Q2.5 **Please report whether the study followed any explanatory framework identified?** Yes/No |  |
| Q2.6 What t**ypes of explanatory framework used the study?**  [Anderson 1995; other] |  |
| Q2.7 **Lack of contact terminology:** forgone care/unmet need/delayed care/other |  |
| Q2.8 **Definition of lack of contact available ?** Yes/no |  |
| **Q2.9 Terminology used in the survey available?** Yes/no |  |
| **Q2.10 Lack of contact indicator type:** complement of an utilization ratio/self-reported/other |  |
| **Q2.11 Disaggregation of lack of contact by type of service or product? Yes/no** |  |
| **Q2.12 Type of service or product considered: outpatient/inpatient/medicines/ health products/other** |  |
| **Q2.13 Identification of the process (barriers) resulting in lack of contact?** Yes/no  *[comment could have said outcome variable related to barriers]* |  |
| **Q2.14 Number of barriers’ dimensions included in the paper** (integer) |  |
| **Q2.15 Type of barrier’s dimension included in the paper (one column per barrier):** Availability/Accessibility/Acceptability/other |  |
| **Q2.16 Identification of financial barriers?** Yes/no |  |
| **Q2.17 Mapping of financial barriers (indicate in which of the four barrier’s dimension financial ones are included)** |  |
| **Q2.18 Number of financial barriers (integer)** |  |
| **Q2.19 Type of financial barriers:** direct /direct/both (direct related to the actual cost of using/getting the service/health product; else classify as indirect) |  |
| **Q2.20. What types of denominator used to estimate proportion?**  **Like adult/children/total adult/etc?** |  |
| **Q.21 Recall period used to collect information about outcome variables. (integer in months)** |  |

Section 3: Please provide outcomes *name* with brief description or definition in the following tables.

| No. | Outcomes | Description/Definition |
| --- | --- | --- |
| 1 |  |  |
| 2 |  |  |
| 3 |  |  |
| 4 |  |  |
| 5 |  |  |
| 6 |  |  |
| 7 |  |  |
| 8 |  |  |

Section 4: Please report the detail reason for unmet needs/forgo care/delayed care

| No. | Reasons | n/N | Percentage |
| --- | --- | --- | --- |
| 1 |  |  |  |
| 2 |  |  |  |
| 3 |  |  |  |
| 4 |  |  |  |
| 5 |  |  |  |
| 6 |  |  |  |
| 7 |  |  |  |
| 8 |  |  |  |
|  |  |  |  |
|  |  |  |  |
|  |  |  |  |

Please use extra rows if required

Section 5: Please report the prevalence of long-term care by country and other characteristics

| Characteristics | n/N | Percentage |
| --- | --- | --- |
|  |  |  |
|  |  |  |
|  |  |  |
|  |  |  |
|  |  |  |
|  |  |  |
|  |  |  |
|  |  |  |
|  |  |  |
|  |  |  |
|  |  |  |

Please use extra rows if required

**Section 6: Prevalence of unmet needs at national and different socio-demographic levels**

|  | Forgo/unmet needs | Delayed care | Others (please specify) |
| --- | --- | --- | --- |
|  | n/N (percent) | n/N (percent) | n/N (percent) |
| National |  |  |  |
| **Residence** |  |  |  |
| Urban |  |  |  |
| Rural |  |  |  |
| **Gender** |  |  |  |
| Male |  |  |  |
| Female |  |  |  |
| **Income categories** |  |  |  |
| Poor |  |  |  |
| Average |  |  |  |
| Rich |  |  |  |
| Insurance status |  |  |  |
| Insured |  |  |  |
| Un-insured |  |  |  |
|  |  |  |  |
|  |  |  |  |
|  |  |  |  |
|  |  |  |  |
|  |  |  |  |
|  |  |  |  |
|  |  |  |  |
|  |  |  |  |
|  |  |  |  |

**[Please present all proportion by all socio-demographic characteristics]**

**Section 7: Please report briefly on each item in the following table**

|  | Author reported |
| --- | --- |
| Objectives |  |
| Participants selection |  |
| Statistical methods |  |
| Unit of analysis |  |
| Results |  |
| Conclusion |  |

**e-method 2: Outcome variables**

The primary outcome of interest was whether the persons was unable to receive treatment or if the person was delayed in receiving treatment. More specifically, people who reported forgoing care, unmet needs or delaying needed medical, dental, or pharmacy care was considered to have delayed or not received necessary care. A typical question generally asked in most of studies about forgone care: “Respondent was asked if, there was a time in the past year they needed any type of (health) care but did not get it”?^1-7^ In case of unmet healthcare needs, is the response to the survey question-“During the past 12 months, was there ever a time when you felt that you needed health care but didn’t receive it?”^8-12^ The reasons for unmet needs or forgo care are the response to the question, “Thinking of the most recent time, why didn’t you get care?”^9-11^ The detailed reasons for unmet needs such as availability (waiting time too long, not available when required, not available in area, etc.), accessibility (cost, transpiration, distance, etc.), and acceptability (felt it would be inadequate, didn’t get around to it, decided not to seek care, too busy, don’t know where to go, dislike doctors/afraid, personal/family responsibilities, etc.) were also included with our listed outcomes. Forgone healthcare or delayed care was treated as unmet needs in this systematic review.

Individuals need long-term care when a chronic condition, trauma, or illness limits their ability to carry out basic self-care tasks. The task are mainly limitations in performing ADLs (including eating, bathing, toileting, and dressing), IADLs (including laundry, shopping for groceries or personal items, meal preparation, banking or paying bills, and keeping track of medication), and mobility tasks (going outside the home, getting around inside the home, and getting out of bed). Generally, respondents were designated as having unmet LTC need if not receiving any help in the prior week, or receiving insufficient help, or having to wait to do the ADL/IADL task because of lack of human help.^13,14^ In most surveys, participants were asked whether they needed any assistance in each ADL/IADL task. Finally, unmet needs for a specific ADL/IADL task had two response categories: having needs unmet (1) and having needs met (0).

**Table S5: Background characteristics of the study (N=87)**

| **Study**  **(Study design)** | **Country (participant)** | **Survey year**  **(Sample size)** | **Type of survey** | **Outcome variables** | **Type of barrier framework (Number)** | **Name of explanatory framework** |
| --- | --- | --- | --- | --- | --- | --- |
| Allen et al., 2017^1^ (Cross) | USA (Adult and Older People) | 2008 (2062) | Health survey | Delayed care, forgone care | N/A (19) | N/A |
| Allin et al., 2010^15^ (Cross) | Canada (Adult and Older People) | 2003 (97828) | Health survey | Unmet needs | N/A (14) | N/A |
| Alonso et al., 2007^16^ (Cross) | Multicounty (Adult and Older People) | 2001-2003 (8796) | Health survey | Unmet needs | N/A (0) | N/A |
| Atanasova et al., 2016^17^ (Cross) | Bulgaria (Adult and Older People) | 2014 (618) | Income and living conditions | Forgone care | N/A (6) | N/A |
| Bagshaw et al., 2017^18^ (Cross) | New Zealand (Adult and Older People) | 2015-2016 (1277) | Health survey | Forgone care, unmet needs | N/A (5) | N/A |
| Bazin et al., 2005^20^ (Cross) | France (Adult and Older People) | 2001-2003 (518) | Income and living conditions | Forgone care | N/A (1) | N/A |
| Bonfrer et al., 2017^21^ (Cross) | Kenya (All) | 2011 (7599) | Household economic and condition survey | Forgone care, forgone checkup/examination, forgone prescription/medications | N/A (10) | N/A |
| Butler et al., 2017^24^ (Cross) | Multicountry (Children) | 2011-2014 (6361) | Health survey | Unmet needs surgical care | N/A (2) | N/A |
| Chae et al., 2017^27^ (Cross) | South Korea (Older People) | 2010 (1419) | Health survey | Unmet needs for dental care | NA (7) | Andersen |
| Chen and Hou., 2002^28^ (Cross) | Canada (Adult and Older People) | 1994-1999 (14143) | Health | Unmet needs | N/A (14) | Andersen |
| Choi et al., 2020^29^ (Cross) | USA (Older People) | 2013-2018 (97309) | Health survey | Unmet needs | N/A (0) | N/A |
| Clark et al., 2016^30^ (Cross) | USA (Adult and Older People) | 2012 (209760) | Health survey | Forgone care | N/A (1) | N/A |
| Cohen et al., 1997^31^ (Cross) | USA (Older People) | 1993 & 2000 (11916) | Income and living conditions | Delayed care, unmet needs | N/A (6) | N/A |
| Connolly and Wren 2017^12^ (Cross) | Ireland (Adolescents, adults, and Older People) | 2013 (9429) | Income and living conditions | Unmet needs | N/A (8) | Andersen |
| Cordasco et al., 2016^32^ (Cross) | USA (Adult and Older People) | 2008-2009 (3600) | Health survey | Delayed/forgone care | N/A (4) | Andersen |
| Corscadden et al., 2019^33^ (Cross) | Multicountry (Adult and Older People) | 2013 and 2016 (3604) | Health survey | Unmet needs for mental health | N/A (5) | Andersen |
| Corscadden et al., 2017^34^ (Cross) | Multicountry (Adult and Older People) | 2013 (20045) | HECS | Forgone checkup/examination, forgone medical visits, forgone prescription/medications | Levesque (11) | Schoen |
| Cunnigham and Hadley 2007^35^ (Cross) | USA (Adult and Older People) | 2003 (37057) | Socio-economic Survey | Unmet needs | N/A (0) | N/A |
| Elofsson et al., 1998^37^ (Cross) | Sweden (Adult and Older People) | 1995 (7929) | Health survey | Forgone care | N/A (1) | N/A |
| Elstad et al., 2016^38^ (Cross) | Multicountry (Adult) | 2008-2013 (1242361) | Income and living conditions | Forgone care | N/A (3) | N/A |
| Fjaer et al., 2017^40^ (Cross) | Multicountry (Adult and Older People) | 2014-2015 (27879) | Socio-economic Survey | Unmet needs | Chen et al (6) | N/A |
| Ford et al., 1999^2^ (Cross) | USA (Adolescents ) | 1995 (12102) | Health survey | Forgone care | N/A (10) | N/A |
| Garcia-Subirats et al., 2014^43^ (Cross) | Multicountry (All) | 2011 (4318) | Health Survey | Forgone care | N/A (13) | O’Halloran |
| Gordon et al., 2020^44^ (Cross) | South Africa (Adolescents, adults, and Older People) | 2012 (25539) | Health survey | Delayed care, unmet needs | Levesque (6) | N/A |
| Guessous et al., 2012^3^ (Cross) | Switzerland (Adult and Older People) | 2007-2010 (2601) | Income and living conditions | Forgone care | N/A (1) | N/A |
| Guessous et al., 2014^47^ (Cross) | Switzerland (Adult and Older People) | 2007-2012 (4313) | Socio-Economic Survey | Forgone dental care | N/A (0) | N/A |
| Ha et al., 2018^48^ (Cross) | South Korea (Adolescents, adults and Older People) | 2012 (3286) | Health survey | Unmet needs | N/A (0) | N/A |
| Hageman et al., 2019^49^ (Cross) | USA (Adult and Older People) | 2015 (12265) | Health survey | Unmet needs | N/A (0) | N/A |
| Hodkinson et al., 2020^53^ (Cross) | South Africa (Children, adolescents, adults and Older People) | 2018 (2754) | Economic Survey | Unmet needs | N/A (0) | N/A |
| Hollederer et al 2019^54^ (Cross) | Germany (Adult and Older People) | 2014 (22695) | Income and living condition survey | Unmet needs, unmet needs for dental care, unmet needs for medical care |  |  |
| Huang et al., 2005^55^ (Cross) | United States (Children and adolescents) | 2001 (38866) | Health Survey | Delayed/ forgone care | N/A (12) | N/A |
| Hwang 2018^8^ (Cross) | South Korea (Adult and Older People) | 2010-2012 (25534) | Health survey | Unmet needs | Chen et al (12) | Andersen |
| Karaca-Mandic et al., 2014^57^ (Cross) | USA (Children) | 2002-2009 (63462) | Household income and expenditure Survey | Unmet needs | N/A (10) | N/A |
| Kim et al., 2017^60^ (Cross) | Multicounty (Adult) | 2011 (44363) | Health and health care | Forgone care | N/A (1) | N/A |
| Klein et al., 2004^61^ (Cross) | United States (Adult and Older People) | 1995-1996 (6535) | Health survey | Delayed care | N/A (1) | N/A |
| Ko 2016^62^ (Cross) | South Korea (Adults and Older People) | 2009-2012 (30868) | Health survey | Unmet needs | N/A (4) | N/A |
| Kotelbova and Soltes 2017^63^ (Cross) | Slovakia (All) | 2013 (145729) | Income and living condition survey | Unmet needs | N/A (8) | N/A |
| Kullgren et al., 2010^64^ (Cross) | USA (All) | 2009 (414) | Health survey | Delayed/ forgone care | N/A (1) | N/A |
| Lee et al., 2016^65^ (Cross) | South Korea (Adult and Older People) | 2012 (228902) | Health survey | Unmet needs | N/A (5) | N/A |
| Lee et al., 2019^66^ (Cross) | South Korea (Older People) | 2017 (63388) | Health survey | Unmet needs | N/A (0) | N/A |
| Lee et al., 2015^67^ (Cross) | South Korea (Adult) | 2008-2009 (9163) | Health survey | Unmet needs | N/A (4) | N/A |
| Levesque et al., 2012^68^ (Cross) | Canada (Adult and Older People) | 2005 (9205) | Health survey | Unmet needs | N/A (8) | N/A |
| Lindstrom et al., 2018^71^ (Cross) | Sweden (Adult and Older People) | 2012 (28029) | Health survey | Unmet needs | N/A (10) | N/A |
| Listl et al., 2016^72^ (Cross) | Multicounty (Older People) | 2004-2005 (13935) | Health survey | Forgone dental care | N/A (1) | N/A |
| Litwin et al., 2009^5^ (Cross) | Multicounty (Older People) | 2004-2005 (28849) | Health survey | Forgone care | N/A (1) | Andersen |
| Lucevic et al., 2019^6^ (Cross) | Hungary (Adult and Older People) | 2019 (1000) | Health survey | Forgone checkup/examination, forgone medical visits, forgone prescription/medications | N/A (2) | N/A |
| Maille et al., 2017^73^ (Cross) | France (Older People) | 2008-2009 (14129) | Health survey | Forgone care | N/A (9) | N/A |
| Marcin et al., 2014^74^ (Cross) | Multicounty (Adolescent, adult, and Older People) | 2005-2009 (574390) | Income and living conditions survey | Unmet needs | N/A (4) | N/A |
| Marshall et al., 2011^75^ (Cross) | Canada (Adolescent, adult, and Older People ) | 2003 (134072) | Health survey | Unmet needs | N/A (0) | Andersen |
| McLeod et al., 2020^76^ (Cross) | Canada (Adult and Older People) | 2014 (52825) | Health survey | Unmet needs | N/A (10) | Andersen |
| Mielck et al., 2009^77^ (Cross) | Multicounty (Older People) | 2004-2005 (14178) | Health survey | Forgone care | N/A (2) | N/A |
| Njagi et al., 2020^79^ (Cross) | Kenya (All) | 2013 (152566) | Health survey | Unmet needs | N/A (9) | Andersen |
| Pathman et al., 2006^80^ (Cross) | Southeastern United States (Adult and Older People) | 2002-2003 (3395) | Health survey | Delayed care, forgone care | N/A (6) | N/A |
| Petrelli et al., 2019^82^ (Cross) | Italy (Adolescent, adult, and Older People) | 2004-2015 (502766) | Income and living conditions survey | Forgone care | N/A (3) | N/A |
| Popovic et al., 2017^83^ (Cross) | Serbia (Adolescent, adult, and Older People) | 2014 (16219) | Income and Living Conditions | Unmet needs | N/A (7) | Andersen |
| Prazeres and Santiago 2016^84^ (Cross) | Portugal (Adult and Older People) | 2014-2015 (521) | Income and living conditions | unmet needs | N/A (8) | N/A |
| Reichard et al. 2017^85^ (Cross) | United States (Adult and Older People) | 2004-2007 (134693) | Income and living conditions | Delayed/ foregone care | N/A (6) | N/A |
| Ro et al., 2017^89^ (Cross) | Cameroon (All) | 2014 (3201) | Health survey | Unmet needs for emergency care | N/A (7) | N/A |
| Ronksley et al., 2012^90^ (Cross) | Canada (Adult and Older People) | 2001,2003, 2005 (360105) | Health survey | Unmet needs | Chen et al (13) | Andersen |
| Samargia et al., 2006^91^ (Cross) | United States (Adolescent) | 2001 (878) | Health survey | Forgone care | N/A (11) | Andersen |
| Sanmartin et al., 2002^92^ (Cross) | Canada (Adolescent, adult, and Older People) | 1994-1999, 2000-2001 (161853) | Health survey | Unmet needs | N/A (13) | N/A |
| Schoen et al., 2013^93^ (Cross) | Multicounty (Adult and Older People) | 2013 (20045) | Health survey | Forgone care, forgone dental care | N/A (1) | N/A |
| Seung et al., 2019^94^ (Cross) | South Korea (Adult) | 2017 (63338) | Health survey | Unmet needs | NA (0) | N/A |
| Shi et al., 2009^95^ (Cross) | United States (Adult) | 2006 (29868) | Health survey | Delayed care, forgone care | N/A (1) | N/A |
| Sibley et al., 2009^9^ (Cross) | Canada (Adult, Older People) | 2003 (111258) | Health survey | Unmet needs | Chen et al (14) | Andersen |
| Smaldone et al., 2005^98^ (Cross) | United States (Children and adolescents) | 2000-2002 (557775) | Health survey | Delayed/ forgone care | N/A (0) | N/A |
| Tambor et al., 2014^101^ (Cross) | Multicountry (All) | 2010 (6052) | Income and living conditions | Forgone care | N/A (0) | N/A |
| Thammatacharee et al., 2012^102^ (Cross) | Thailand (All) | 2010 (5469) | Socio-Economic Survey | Unmet needs | N/A (8) | N/A |
| Towne 2017^103^ (Cross) | USA (Adult and Older People) | 2011–2015 (2380047) | Health and living condition survey | Forgone care | N/A (1) | N/A |
| Washington et al., 2011^106^ (Cross) | United States (Adult and Older People) | 2008-2009 (3611) | Health and living condition survey | Unmet needs | N/A (4) | Andersen |
| Weathers et al., 2004^107^ (Cross) | United States (Child) | 1999 (300) | Health survey | Unmet needs | N/A (13) | Andersen |
| Wiltshire et al., 2009^108^ (Cross) | United States (Adult, Older People) | 2003-2004 (18404) | Health survey | Unmet needs | N/A (0) | N/A |
| Wisk et al., 2014^110^ (Cross) | United States (Adult) | 2001-2008 (14138) | Health survey | Unmet needs | N/A (2) | Andersen |
| Wu et al., 2005^111^ (Cross) | Canada (Adult) | 2000-2001 (118219) | Health survey | Unmet needs | N/A (14) | Andersen |
| Yamada et al., 2015^112^ (Cross) | USA (Older People) | 2003-2004 (17797) | Household survey | Unmet needs | N/A (4) | N/A |
| Yu et al., 2008^113^ (Cross) | United States (Young Adulthood) | 1995, 2001-2002 (10817) | Health survey | Forgone mental care | N/A (15) | Andersen |
| Zhu 2015^115^ (Cross) | China (Older People) | 2005, 2008, and 2011 (7504) | Health survey | Unmet needs | N/A (4) | N/A |
| Zhu and Osterle 2017^116^ (Cross) | China (Older People) | 2013 (8558) | Health survey | Unmet needs | N/A (8) | N/A |
| Zimmer et al., 1997^117^ (Cross) | Oregon, United States (High School Students) | 1995 (13992) | Health survey | Unmet needs | N/A (5) | N/A |

Cross, cross-section study

**Table S6: Background characteristics of unmet long-term care needs study (N=14)**

| Study (Study design) | Survey year (N) | Country | Population (age, years) | Outcome | Caregiver |
| --- | --- | --- | --- | --- | --- |
| Abrahamson et al., 2017^118^ (Long) | 2010 (1352) | USA | ADL-subjects  (67-104) | unmet LTC | Any |
| Andrade et al., 2018^119^ (Cross) | 2013 (2165) | Brazil | ADL-subjects  (≥60) | unmet LTC | Family member,  hired caregiver/maid |
| Andrade et al., 2018^119^ (cross) | 2013 (5762) | Brazil | ADL-subjects  (≥60) | unmet LTC | Family member,  hired caregiver/maid |
| Fu et al., 2017^120^ (Long) | 2014 (1090) | China | IADL-subjects  (≥60) | unmet LTC | Any  (family/HCBC/institutional care) |
| Hass et al., 2017^121^ (Cohort) | 2005 (2194) | USA | ADL-subjects  (≥65) | unmet LTC | Institutional care |
| He et al., 2015^122^ (Cohort) | 1994-2004 (7228) | USA | ADL-subjects  (≥65) | unmet LTC | Any |
| Hledec et al., 2016^123^ (Cross) | 2013 (1458) | Slovenian | ADL-subjects  (≥65) | unmet LTC | Any |
| Hu et al., 2018^124^ (Long) | 2014 (1324) | China | ADL-subjects  (≥60) | unmet LTC | Any |
| Li 2006^125^ (Cross) | 1999 (278) | USA | ADL/IADL-subjects  (≥65) | unmet LTC | Any |
| Tennstedt et al., 1994^126^ (Long) | 1984 (235) | USA | ADL/IADL//PADL-subjects  (≥70) | Unmet LTC | Non-paid friends and  family member |
| Xiang et al., 2018^127^ (Cross) | 2015 (3361) | USA | ADL/IADL-subjects  (≥65) | Unmet LTC | Any |
| Zhen et al., 2015^128^ (Long) | 2005 (3089) | China | ADL-subjects  (≥65) | unmet LTC | Any |
| Zhu., 2015^115^ (Long) | 2005 (2938); 2008 (2919);  2011 (1647) | China | ADL/IADL-subjects  (80-109) | unmet LTC | Any |
| Zuverink et al., 2020^129^ (Long) | 2011-2016 (3936) | USA | ADL/IADL-subjects  (≥65) | unmet LTC | Any |

ADL, activities of daily living; IADL, instrumental activities of daily living; Cross, Cross-sectional study; Cohort, Prospective cohort; Long, Longitudinal Survey; N, LTC, long-term care; total sample size; USA, United State of America

Table S7: Quality assessment of the cross-sectional studies to unmet needs

| Author | **Selection (5 points)** |  |  |  |  | **Comparability (2 points)** |  |  | **Outcome (3 points)** |  | Total Score |
| --- | --- | --- | --- | --- | --- | --- | --- | --- | --- | --- | --- |
|  | 1 | 2 | 3 | 4 |  | 5A | 5B |  | 6 | 7 |  |
|  | Representativeness of the sample (*) | Sample size (*) | Non-respondents (*) | Ascertainment of the exposure ** |  | Controls for age (*) | Control for any additional factor (*) |  | Assessment of the outcome (**) | Statistical test (*) |  |
| Calzon (Fernández et al) 2015 | 1 | 1 | 0 | 2 |  | 1 | 1 |  | 1 | 1 | 8 |
| Allen et al. (2017) | 1 | 1 | 1 | 1 |  | 1 | 1 |  | 1 | 1 | 8 |
| Allin et al. (2010) | 1 | 1 | 1 | 1 |  | 0 | 1 |  | 1 | 1 | 7 |
| Alonso (2007) | 1 | 1 | 0 | 1 |  | 0 | 0 |  | 1 | 1 | 5 |
| Atanasova et al. (2016) | 1 | 1 | 0 | 1 |  | 0 | 1 |  | 1 | 0 | 5 |
| Bagshaw et al. (2017) | 1 | 1 | 1 | 1 |  | 0 | 1 |  | 1 | 1 | 7 |
| Baughman et al. (2015) | 1 | 1 | 0 | 2 |  | 1 | 1 |  | 1 | 1 | 8 |
| Bonfrer et al. (2017) | 0 | 1 | 1 | 0 |  | 0 | 0 |  | 1 | 1 | 4 |
| Bremer (2014) | 1 | 1 | 0 | 1 |  | 1 | 1 |  | 1 | 1 | 7 |
| Burgard et al. (2014) | 1 | 1 | 0 | 2 |  | 1 | 1 |  | 1 | 1 | 8 |
| Butler et al. (2017) | 1 | 1 | 1 | 2 |  | 1 | 1 |  | 1 | 1 | 9 |
| Cavalieri (2013) | 1 | 1 | 0 | 2 |  | 1 | 1 |  | 1 | 1 | 8 |
| CDC (2010) | 1 | 0 | 0 | 1 |  | 1 | 1 |  | 1 | 1 | 6 |
| Chae et al. (2017) | 1 | 1 | 0 | 1 |  | 1 | 1 |  | 1 | 1 | 7 |
| Chaupain-Guillot and Guillot (2014) | 1 | 1 | 0 | 1 |  | 1 | 1 |  | 1 | 1 | 7 |
| Chen and Hou (2002) | 1 | 1 | 1 | 1 |  | 1 | 1 |  | 1 | 1 | 8 |
| Choi et al. (2020) | 1 | 1 | 0 | 1 |  | 1 | 1 |  | 1 | 1 | 7 |
| Clark et al. (2016) | 1 | 1 | 0 | 1 |  | 0 | 1 |  | 1 | 1 | 6 |
| Cohen et al. (1997) | 1 | 1 | 0 | 1 |  | 1 | 1 |  | 1 | 1 | 7 |
| Connolly and Wren (2017) | 1 | 1 | 0 | 1 |  | 1 | 1 |  | 1 | 1 | 7 |
| Cordasco et al. (2016) | 1 | 1 | 0 | 1 |  | 1 | 1 |  | 1 | 1 | 7 |
| Corscadden et al. (2017) | 1 | 1 | 1 | 1 |  | 0 | 0 |  | 1 | 0 | 5 |
| Corscadden et al. (2019) | 1 | 1 | 0 | 0 |  | 1 | 1 |  | 1 | 1 | 6 |
| Cunnigham and Hadley (2007) | 1 | 1 | 0 | 1 |  | 0 | 1 |  | 1 | 1 | 6 |
| Denny et al. (2013) | 1 | 1 | 1 | 1 |  | 1 | 1 |  | 1 | 1 | 8 |
| Elofsson et al. (1998) | 1 | 1 | 0 | 2 |  | 1 | 1 |  | 2 | 1 | 9 |
| Fjaer et al. (2017) | 1 | 1 | 0 | 2 |  | 1 | 1 |  | 1 | 1 | 8 |
| Footman et al. (2014) | 1 | 1 | 0 | 2 |  | 1 | 1 |  | 1 | 1 | 8 |
| Gordon et al. (2020) | 1 | 1 | 1 | 2 |  | 1 | 1 |  | 1 | 1 | 9 |
| Griffin-Tomas et al. (2019) | 1 | 1 | 0 | 1 |  | 1 | 1 |  | 1 | 1 | 7 |
| Guessous et al. (2014) | 1 | 1 | 0 | 1 |  | 1 | 1 |  | 1 | 1 | 7 |
| Ha et al. (2018) | 1 | 1 | 0 | 2 |  | 1 | 1 |  | 1 | 1 | 8 |
| Hageman et al. (2019) | 1 | 1 | 0 | 2 |  | 1 | 1 |  | 1 | 1 | 8 |
| Heck and Parker (2002) | 1 | 1 | 1 | 1 |  | 1 | 1 |  | 1 | 1 | 8 |
| Hodkinson et al. (2019) | 1 | 1 | 1 | 1 |  | 1 | 1 |  | 1 | 1 | 8 |
| Kalousova and Burgard (2013) | 1 | 1 | 1 | 1 |  | 0 | 1 |  | 1 | 1 | 7 |
| Kaye (2019) | 1 | 1 | 0 | 1 |  | 1 | 1 |  | 1 | 1 | 7 |
| Kim et al. (2017) | 1 | 1 | 0 | 1 |  | 1 | 1 |  | 1 | 1 | 7 |
| Kotelbova and Soltes (2017) | 1 | 1 | 0 | 1 |  | 0 | 0 |  | 1 | 1 | 5 |
| Kullgren et al. (2010) | 1 | 1 | 0 | 1 |  | 1 | 1 |  | 1 | 1 | 7 |
| Lee et al. (2015) | 1 | 0 | 0 | 1 |  | 1 | 1 |  | 1 | 1 | 6 |
| Lee et al. (2016) | 0 | 1 | 1 | 0 |  | 1 | 1 |  | 1 | 1 | 6 |
| Lee et al. (2019) | 0 | 1 | 0 | 0 |  | 1 | 1 |  | 1 | 1 | 5 |
| Levesque et al. (2012) | 1 | 1 | 0 | 1 |  | 1 | 1 |  | 1 | 1 | 7 |
| Li et al. (2018) | 1 | 0 | 0 | 1 |  | 1 | 1 |  | 1 | 1 | 6 |
| Lindstrome et al. (2017) | 1 | 0 | 0 | 1 |  | 1 | 1 |  | 1 | 1 | 6 |
| Lindstrome et al. (2017) | 1 | 0 | 0 | 1 |  | 1 | 1 |  | 1 | 1 | 6 |
| Listl et al. (2016) | 1 | 0 | 0 | 1 |  | 1 | 1 |  | 1 | 1 | 6 |
| Litwin et al. (2009) | 1 | 0 | 1 | 1 |  | 1 | 1 |  | 1 | 1 | 7 |
| Lucevic et al. (2019) | 1 | 0 | 1 | 1 |  | 1 | 1 |  | 1 | 1 | 7 |
| Maille et al. (2017) | 1 | 0 | 0 | 1 |  | 0 | 0 |  | 1 | 0 | 3 |
| Marshall et al. (2011) | 1 | 0 | 1 | 1 |  | 1 | 1 |  | 1 | 1 | 7 |
| Mielck et al. (2009) | 0 | 0 | 0 | 1 |  | 1 | 1 |  | 1 | 1 | 5 |
| Newacheck et al. (2000) | 1 | 0 | 1 | 1 |  | 1 | 1 |  | 1 | 1 | 7 |
| Njagi et al. (2020) | 1 | 0 | 1 | 1 |  | 1 | 1 |  | 1 | 1 | 7 |
| Pathman et al. (2006) | 0 | 0 | 0 | 0 |  | 1 | 1 |  | 1 | 1 | 4 |
| Peterson et al. (2010) | 1 | 0 | 0 | 0 |  | 1 | 1 |  | 1 | 1 | 5 |
| Peterson et al. (2010) | 1 | 0 | 0 | 0 |  | 1 | 1 |  | 1 | 1 | 5 |
| Petrelli et al. (2019) | 1 | 0 | 1 | 1 |  | 1 | 1 |  | 1 | 1 | 7 |
| Popovic et al. (2017) | 1 | 0 | 1 | 1 |  | 1 | 1 |  | 1 | 1 | 7 |
| Rew et al. (1999) | 1 | 0 | 1 | 1 |  | 0 | 1 |  | 1 | 1 | 6 |
| Rhee et al. (2019) | 0 | 0 | 0 | 1 |  | 1 | 1 |  | 1 | 1 | 5 |
| Riverbark et al. (2020) | 1 | 0 | 0 | 1 |  | 1 | 1 |  | 1 | 1 | 6 |
| Ro et al. (2017) | 0 | 1 | 1 | 0 |  | 1 | 1 |  | 1 | 1 | 6 |
| Ronksley et al. (2012) | 1 | 0 | 0 | 1 |  | 1 | 1 |  | 1 | 1 | 6 |
| Samargia et al. (2006) | 0 | 0 | 1 | 1 |  | 0 | 1 |  | 1 | 1 | 5 |
| Sanmartin et al. (2002) | 0 | 0 | 1 | 1 |  | 1 | 1 |  | 1 | 1 | 6 |
| Seung et al. (2019) | 1 | 0 | 0 | 1 |  | 1 | 1 |  | 1 | 1 | 6 |
| Shi et al. (2005) | 1 | 0 | 0 | 1 |  | 1 | 1 |  | 1 | 1 | 6 |
| Shi et al. (2009) | 1 | 0 | 0 | 1 |  | 0 | 1 |  | 1 | 1 | 5 |
| Sibley et al. (2009) | 0 | 1 | 0 | 2 |  | 1 | 1 |  | 1 | 0 | 6 |
| Silver et al. (2001) | 1 | 0 | 1 | 1 |  | 1 | 1 |  | 1 | 0 | 6 |
| Stein et al. (2019) | 1 | 1 | 0 | 2 |  | 0 | 0 |  | 1 | 1 | 6 |
| Stransky et al. (2018) | 1 | 1 | 0 | 0 |  | 0 | 1 |  | 1 | 1 | 5 |
| Thammatacharee et al. (2012) | 1 | 1 | 0 | 1 |  | 0 | 0 |  | 1 | 1 | 5 |
| Towne (2017) | 1 | 0 | 0 | 0 |  | 0 | 1 |  | 1 | 1 | 4 |
| Travers et al. (2017) | 1 | 0 | 0 | 0 |  | 1 | 1 |  | 1 | 1 | 5 |
| Washington et al. (2011) | 1 | 1 | 1 | 0 |  | 1 | 1 |  | 1 | 1 | 7 |
| Weathers et al. (2004) | 0 | 1 | 0 | 1 |  | 1 | 1 |  | 1 | 1 | 6 |
| Wiltshire et al. (2009) | 1 | 1 | 0 | 2 |  | 1 | 1 |  | 1 | 1 | 8 |
| Wu et al. (2005) | 1 | 1 | 0 | 2 |  | 0 | 1 |  | 0 | 1 | 6 |
| Yamad et al. (2015) | 1 | 1 | 0 | 0 |  | 0 | 1 |  | 1 | 1 | 5 |
| Yu et al. (2008) | 1 | 1 | 0 | 0 |  | 0 | 0 |  | 1 | 1 | 4 |
| Zhu (2015) | 0 | 0 | 1 | 0 |  | 0 | 1 |  | 0 | 1 | 3 |
| Zhu and Osterle (2017) | 1 | 0 | 0 | 0 |  | 1 | 1 |  | 1 | 1 | 5 |
| Zimmer et al. (1997) | 0 | 0 | 0 | 0 |  | 1 | 1 |  | 1 | 1 | 4 |

High if scored ≥6, moderate if they scored 4–5, and low if they scored 0–3

Table S8: Quality assessment of the Cohort studies related to unmet needs

| Author | **Selection** | | | |  | **Comparability** | |  | **Outcome** | | | Total score |
| --- | --- | --- | --- | --- | --- | --- | --- | --- | --- | --- | --- | --- |
|  | 1 | 2 | 3 | 4 |  | 5A | 5B |  | 6 | 7 | 8 |  |
|  | Exposed cohort truly representative | Non-exposed cohort drawn from the same community | Ascertainment of exposure | Outcome of interest not present at start |  | Cohorts comparable on basis of age | Cohorts comparable on other factor(s) |  | Quality of outcome assessment | Follow-up long enough for outcomes to occur | Complete accounting for cohorts |  |
| Feral-Pierssens et al. (2020) | 1 | 1 | 0 | 1 |  | 1 | 1 |  | 1 | 1 | 0 | 7 |
| Heard-Garris et al. (2018) | 1 | 1 | 0 | 0 |  | 0 | 1 |  | 1 | 1 | 0 | 5 |
| Karaca-Mandic et al. (2014) | 1 | 1 | 0 | 0 |  | 1 | 1 |  | 1 | 1 | 0 | 6 |
| Ko (2016) | 1 | 1 | 1 | 0 |  | 1 | 1 |  | 1 | 1 | 0 | 7 |
| Winkelman et al (2019) | 1 | 0 | 0 | 1 |  | 1 | 1 |  | 0 | 1 | 0 | 5 |
| Wisk et al (2012) | 1 | 0 | 0 | 1 |  | 1 | 1 |  | 0 | 1 | 0 | 5 |
| Wisk et al (2014) | 1 | 0 | 0 | 1 |  | 1 | 1 |  | 0 | 1 | 0 | 5 |

High if scored ≥6, moderate if they scored 4–5, and low if they scored 0–3

Table S9: Quality assessment of the Cross-sectional studies related to long-term care

| Author | **Selection (5 points)** |  |  |  |  | **Comparability (2 points)** |  |  | **Outcome (3 points)** |  |  |
| --- | --- | --- | --- | --- | --- | --- | --- | --- | --- | --- | --- |
|  | 1 | 2 | 3 | 4 |  | 5A | 5B |  | 6 | 7 |  |
|  | Representativeness of the sample (*) | Sample size (*) | Non-respondents (*) | Ascertainment of the exposure ** |  | Controls for age (*) | Control for any additional factor (*) |  | Assessment of the outcome (**) | Statistical test (*) | Total Score |
| Andrade et al. (2018) | 1 | 0 | 0 | 1 |  | 1 | 1 |  | 2 | 1 | 7 |
| Hledec et al. (2016) | 1 | 0 | 0 | 1 |  | 1 | 1 |  | 1 | 1 | 6 |
| Li (2006) | 0 | 1 | 0 | 0 |  | 1 | 1 |  | 1 | 1 | 5 |
| Xiang (2018) | 1 | 1 | 0 | 0 |  | 1 | 1 |  | 1 | 1 | 6 |

High if scored ≥6, moderate if they scored 4–5, and low if they scored 0–3

Table S10: Quality assessment of the Cohort-sectional studies related to long-term care

| Author | **Selection** | | | |  | **Comparability** | |  | **Outcome** | | | Total score |
| --- | --- | --- | --- | --- | --- | --- | --- | --- | --- | --- | --- | --- |
|  | 1 | 2 | 3 | 4 |  | 5A | 5B |  | 6 | 7 | 8 |  |
|  | Exposed cohort truly representative | Non-exposed cohort drawn from the same community | Ascertainment of exposure | Outcome of interest not present at start |  | Cohorts comparable on basis of age | Cohorts comparable on other factor(s) |  | Quality of outcome assessment | Follow-up long enough for outcomes to occur | Complete accounting for cohorts |  |
| Abrahamson et al. (2017) | 1 | 0 | 0 | 1 |  | 1 | 1 |  | 1 | 0 | 0 | 5 |
| Fu et al. (2017) | 1 | 0 | 1 | 1 |  | 1 | 1 |  | 1 | 0 | 0 | 6 |
| Hass et al. (2017) | 1 | 0 | 0 | 1 |  | 1 | 1 |  | 1 | 0 | 0 | 5 |
| He et al. (2015) | 1 | 0 | 1 | 1 |  | 1 | 1 |  | 1 | 1 | 1 | 8 |
| Hu and Wang, (2018) | 1 | 1 | 1 | 0 |  | 1 | 1 |  | 1 | 1 | 0 | 7 |
| Tennstedt et al. (1994) | 1 | 1 | 1 | 0 |  | 1 | 1 |  | 1 | 1 | 0 | 7 |
| Zhen et al. (2015) | 1 | 1 | 1 | 0 |  | 1 | 1 |  | 1 | 1 | 1 | 8 |
| Zhu (2015) | 1 | 1 | 1 | 0 |  | 1 | 1 |  | 1 | 1 | 1 | 8 |
| Zuverink (2019) | 1 | 1 | 1 | 0 |  | 1 | 1 |  | 1 | 1 | 0 | 7 |

High if scored ≥6, moderate if they scored 4–5, and low if they scored 0–3


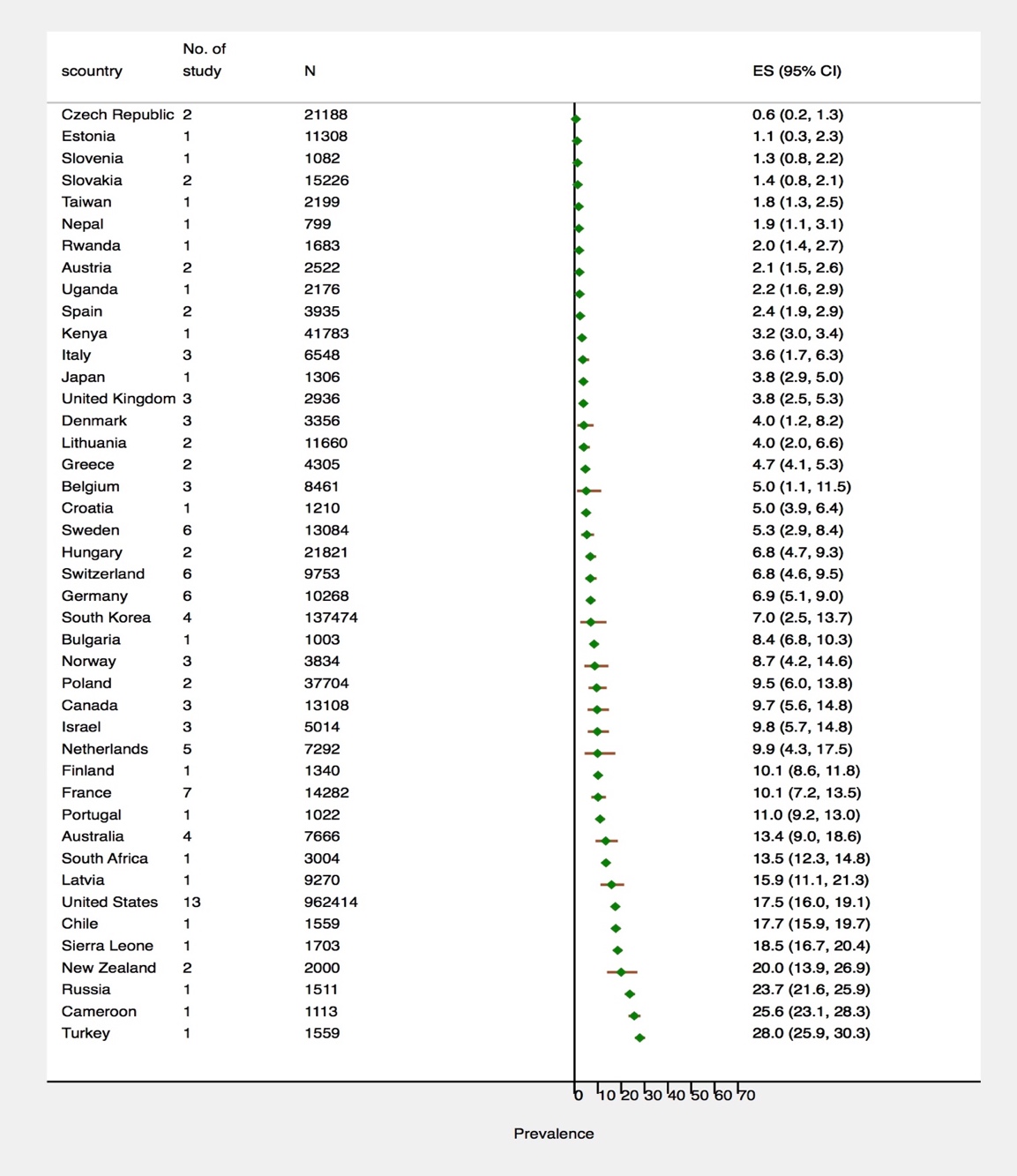


Figure S1: Country-specific prevalence for forgone healthcare due to cost-related reasons among older people, 65 years and above


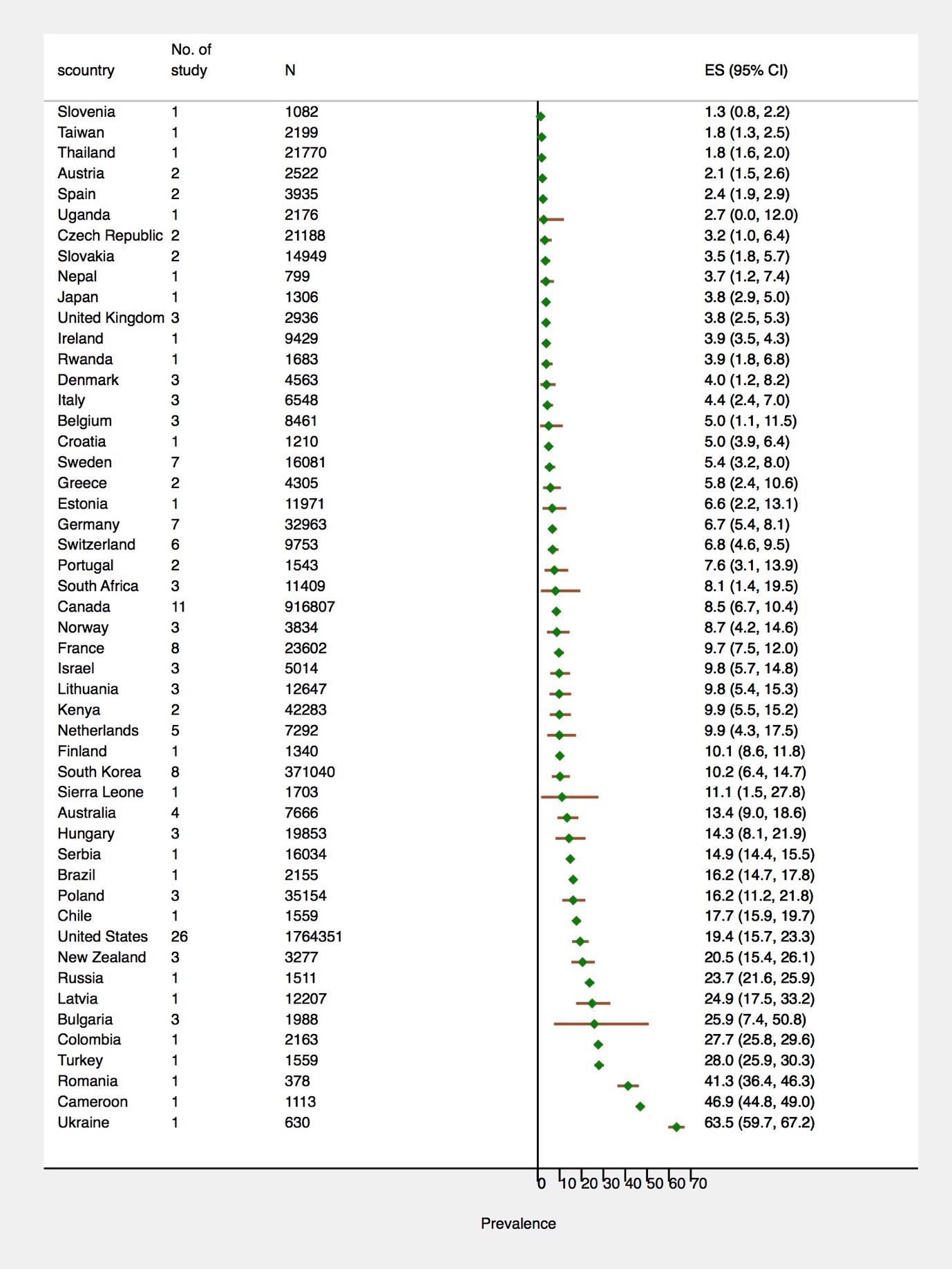


Figure S2: Unmet needs for healthcare among older people due to any reason by country

**References**

1. Allen EM, Call KT, Beebe TJ, McAlpine DD, Johnson PJ. Barriers to Care and Health Care Utilization among the Publicly Insured. *Medical Care* 2017; **55**(3): 207-14.

2. Ford CA, Bearman PS, Moody J. Foregone health care among adolescents. *JAMA* 1999; **282**(23): 2227-34.

3. Guessous I, Gaspoz JMT, Theler JM, Wolff H. High prevalence of forgoing healthcare for economic reasons in Switzerland: a population-based study in a region with universal health insurance coverage. *Prev Med* 2012; **55**(5): 521-7.

4. Guessous I, Theler JM, Durosier Izart C, et al. Forgoing dental care for economic reasons in Switzerland: a six-year cross-sectional population-based study. *BMC Oral Health* 2014; **14**: 121.

5. Litwin H, Sapir EV. Forgone health care due to cost among older adults in European countries and in Israel. *Eur J Ageing* 2009; **6**(3): 167-76.

6. Lucevic A, Péntek M, Kringos D, et al. Unmet medical needs in ambulatory care in Hungary: forgone visits and medications from a representative population survey. *Eur J Health Econ* 2019; **20**: 71-8.

7. Wisk LE, Witt WP. Predictors of delayed or forgone needed health care for families with children. *Pediatrics* 2012; **130**(6): 1027-37.

8. Hwang J. Understanding reasons for unmet health care needs in Korea: what are health policy implications? *BMC Health Serv Res* 2018; **18**(1): 557.

9. Sibley LM, Glazier RH. Reasons for self-reported unmet healthcare needs in Canada: A population-based provincial comparison. *Healthcare Policy* 2009; **5**(1): 87-101.

10. Cavalieri M. Geographical variation of unmet medical needs in Italy: a multivariate logistic regression analysis. *Int J Health Geogr* 2013; **12**: 27.

11. Chaupain-Guillot S, Guillot O. Health system characteristics and unmet care needs in Europe: an analysis based on EU-SILC data. *Health Econ* 2014.

12. Connolly S, Wren MA. Unmet healthcare needs in Ireland: Analysis using the EU-SILC survey. *Health Policy* 2017; **121**(4): 434-41.

13. Hass Z, DePalma G, Craig BA, Xu H, Sands LP. Unmet need for help with activities of daily living disabilities and emergency department admissions among older medicare recipients. *The Gerontologist* 2017; **57**(2): 206-10.

14. Williams J, Lyons B, Rowland D. Unmet long-term care needs of elderly people in the community: a review of the literature. *Home Health Care Serv Q* 1997; **16**(1-2): 93-119.

15. Allin S, Grignon M, Le Grand J. Subjective unmet need and utilization of health care services in Canada: what are the equity implications? *Soc Sci Med* 2010; **70**(3): 465-72.

16. Alonso J, Codony M, Kovess V, et al. Population level of unmet need for mental healthcare in Europe. *British Journal of Psychiatry* 2007; **190**: 299-306.

17. Atanasova E, Rohova M, Dimova A. Unmet needs for health care services in Bulgaria. *Journal of IMAB - Annual Proceeding (Scientific Papers)* 2016; **22**(3): 1324-7.

18. Bagshaw P, Bagshaw S, Frampton C, et al. Pilot study of methods for assessing unmet secondary health care need in New Zealand. *N Z Med J* 2017; **130**(1452): 23-38.

19. Baughman KR, Burke RC, Hewit MS, Sudano JJ, Meeker J, Hull SK. Associations between Difficulty Paying Medical Bills and Forgone Medical and Prescription Drug Care. *Popul Health Manag* 2015; **18**(5): 358-66.

20. Bazin F, Parizot I, Chauvin P. Original approach to the individual characteristics associated with forgone healthcare: a study in underprivileged areas, Paris region, France, 2001-2003. *Eur J Public Health* 2005; **15**(4): 361-7.

21. Bonfrer I, Gustafsson-Wright E. Health shocks, coping strategies and foregone healthcare among agricultural households in Kenya. *Glob Public Health* 2017; **12**(11): 1369-90.

22. Bremer P. Forgone care and financial burden due to out-of-pocket payments within the German health care system. *Health Econ Rev* 2014; **4**(1): 36.

23. Burgard SA, Hawkins JM. Race/Ethnicity, educational attainment, and foregone health care in the United States in the 2007-2009 recession. *Am J Public Health* 2014; **104**(2): e134-40.

24. Butler EK, Tran TM, Nagarajan N, et al. Epidemiology of pediatric surgical needs in low-income countries. *PLoS One* 2017; **12**(3): e0170968.

25. Calzón Fernández S, Fernández Ajuria A, Martín JJ, Murphy MJ. The impact of the economic crisis on unmet dental care needs in Spain. *J Epidemiol Community Health* 2015; **69**(9): 880-5.

26. CDC. Vital signs: health insurance coverage and health care utilization --- United States, 2006--2009 and January-March 2010. *MMWR Morb Mortal Wkly Rep* 2010; **59**(44): 1448-54.

27. Chae S, Lee Y, Kim J, Chun KH, Lee JK. Factors associated with perceived unmet dental care needs of older adults. *Geriatr Gerontol Int* 2017; **17**(11): 1936-42.

28. Chen J, Hou F. Unmet needs for health care. *Health reports / Statistics Canada, Canadian Centre for Health Information = Rapports sur la santé / Statistique Canada, Centre canadien d'information sur la santé* 2002; **13**(2): 23-34.

29. Choi NG, Dinitto DM, Choi BY. Unmet healthcare needs and healthcare access gaps among uninsured U.S. adults aged 50–64. *Int J Environ Res Public Health* 2020; **17**(8).

30. Clark CR, Ommerborn MJ, B AC, Pham do Q, Haas JS. Income Inequities and Medicaid Expansion are Related to Racial and Ethnic Disparities in Delayed or Forgone Care Due to Cost. *Med Care* 2016; **54**(6): 555-61.

31. Cohen RA, Bloom B, Simpson G, Parsons PE. Access to health care. Part 3: Older adults. *Vital Health Stat 10* 1997; (198): 1-32.

32. Cordasco KM, Mengeling MA, Yano EM, Washington DL. Health and Health Care Access of Rural Women Veterans: Findings From the National Survey of Women Veterans. *J Rural Health* 2016; **32**(4): 397-406.

33. Corscadden L, Callander EJ, Topp SM. Who experiences unmet need for mental health services and what other barriers to accessing health care do they face? Findings from Australia and Canada. *Int J Health Plann Manage* 2019; **34**(2): 761-72.

34. Corscadden L, Levesque J-F, Lewis V, et al. Barriers to accessing primary health care: comparing Australian experiences internationally. *Australian Journal of Primary Health* 2017; **23**(3): 223-8.

35. Cunningham PJ, Hadley J. Differences between symptom-specific and general survey questions of unmet need in measuring insurance and racial/ethnic disparities in access to care. *Med Care* 2007; **45**(9): 842-50.

36. Denny S, Farrant B, Cosgriff J, et al. Forgone health care among secondary school students in New Zealand. *J Prim Health Care* 2013; **5**(1): 11-8.

37. Elofsson S, Undén AL, Krakau I. Patient charges--a hindrance to financially and psychosocially disadvantage groups seeking care. *Soc Sci Med* 1998; **46**(10): 1375-80.

38. Elstad JI. Income inequality and foregone medical care in Europe during The Great Recession: multilevel analyses of EU-SILC surveys 2008-2013. *Int J Equity Health* 2016; **15**(1): 101.

39. Feral-Pierssens AL, Rives-Lange C, Matta J, et al. Forgoing health care under universal health insurance: the case of France. *International Journal of Public Health*.

40. Fjaer EL, Stornes P, Borisova LV, McNamara CL, Eikemo TA. Subjective perceptions of unmet need for health care in Europe among social groups: Findings from the European social survey (2014) special module on the social determinants of health. *European Journal of Public Health* 2017; **27**: 82-9.

41. Footman K, Richardson E, Roberts B, et al. Foregoing medicines in the former Soviet Union: changes between 2001 and 2010. *Health Policy* 2014; **118**(2): 184-92.

42. Franks P, Gold MR, Clancy CM. Use of care and subsequent mortality: the importance of gender. *Health Serv Res* 1996; **31**(3): 347-63.

43. Garcia-Subirats I, Vargas I, Mogollon-Perez AS, et al. Barriers in access to healthcare in countries with different health systems. A cross-sectional study in municipalities of central Colombia and north-eastern Brazil. *Soc Sci Med* 2014; **106**: 204-13.

44. Gordon T, Booysen F, Mbonigaba J. Socio-economic inequalities in the multiple dimensions of access to healthcare: the case of South Africa. *BMC Public Health* 2020; **20**(1): 289.

45. Griffin-Tomas M, Cahill S, Kapadia F, Halkitis PN. Access to Health Services Among Young Adult Gay Men in New York City. *Am J Mens Health* 2019; **13**(1): 1557988318818683.

46. Griffin M, Jaiswal J, Krytusa D, Krause KD, Kapadia F, Halkitis PN. Healthcare experiences of urban young adult lesbians. *Womens Health (Lond)* 2020; **16**: 1745506519899820.

47. Guessous I, Theler J-M, Izart CD, et al. Forgoing dental care for economic reasons in Switzerland: a six-year cross-sectional population-based study. *BMC Oral Health* 2014; **14**(1): 121.

48. Ha R, Jung-Choi K, Kim CY. Employment Status and Self-Reported Unmet Healthcare Needs among South Korean Employees. *Int J Environ Res Public Health* 2018; **16**(1).

49. Hageman SA, St George DMM. Health Savings Account Ownership and Financial Barriers to Health Care: What Social Workers Should Know. *Social work in public health* 2019; **34**(2): 176-88.

50. Han X, Call KT, Pintor JK, Alarcon-Espinoza G, Simon AB. Reports of insurance-based discrimination in health care and its association with access to care. *Am J Public Health* 2015; **105 Suppl 3**: S517-25.

51. Heard-Garris N, Winkelman TNA, Choi H, et al. Health Care Use and Health Behaviors Among Young Adults With History of Parental Incarceration. *Pediatrics* 2018; **142**(3).

52. Heck KE, Parker JD. Family structure, socioeconomic status, and access to health care for children. *Health Serv Res* 2002; **37**(1): 173-86.

53. Hodkinson PW, Pigoga JL, Wallis L. Emergency healthcare needs in the Lavender Hill suburb of Cape Town, South Africa: a cross-sectional, community-based household survey. *BMJ Open* 2020; **10**(1): e033643.

54. Hollederer A, Wildner M. [Unmet Medical Need in Germany: Analyses of EU-SILC-Survey from 2005 to 2014]. *Dtsch Med Wochenschr* 2019; **144**(1): e1-e11.

55. Huang ZJ, Kogan MD, Stella MY, Strickland B. Delayed or forgone care among children with special health care needs: an analysis of the 2001 National Survey of Children with Special Health Care Needs. *Ambulatory Pediatrics* 2005; **5**(1): 60-7.

56. Kalousova L, Burgard SA. Debt and foregone medical care. *J Health Soc Behav* 2013; **54**(2): 204-20.

57. Karaca-Mandic P, Choi-Yoo SJ, Lee J, Scal P. Family out-of-pocket health care burden and children's unmet needs or delayed health care. *Acad Pediatr* 2014; **14**(1): 101-8.

58. Kaye HS. Disability-Related Disparities in Access to Health Care Before (2008-2010) and After (2015-2017) the Affordable Care Act. *American journal of public health* 2019; **109**(7): 1015-21.

59. Kerfeld CI, Hoffman JM, Ciol MA, Kartin D. Delayed or forgone care and dissatisfaction with care for children with special health care needs: the role of perceived cultural competency of health care providers. *Matern Child Health J* 2011; **15**(4): 487-96.

60. Kim TJ, Vonneilich N, Lüdecke D, von dem Knesebeck O. Income, financial barriers to health care and public health expenditure: A multilevel analysis of 28 countries. *Social Science and Medicine* 2017; **176**: 158-65.

61. Klein D, Turvey C, Wallace R. Elders who delay medication because of cost: health insurance, demographic, health, and financial correlates. *The Gerontologist* 2004; **44**(6): 779-87.

62. Ko H. Unmet healthcare needs and health status: Panel evidence from Korea. *Health Policy* 2016; **120**(6): 646-53.

63. Kotlebova E, Soltes E, Papiez M, Smiech S. Bayesian estimation of proportions in the analysis of unavailability of health care in Slovakia; 2017.

64. Kullgren JT, Galbraith AA, Hinrichsen VL, et al. Health care use and decision making among lower-income families in high-deductible health plans. *Arch Intern Med* 2010; **170**(21): 1918-25.

65. Lee SE, Yeon M, Kim CW, Yoon TH. The Association Among Individual and Contextual Factors and Unmet Healthcare Needs in South Korea: A Multilevel Study Using National Data. *J Prev Med Public Health* 2016; **49**(5): 308-22.

66. Lee SE, Yeon M, Kim CW, Yoon TH, Kim D, Choi J. Neighborhood deprivation and unmet health care needs: A multilevel analysis of older individuals in South Korea. *Osong Public Health and Research Perspectives* 2019; **10**(5): 295-306.

67. Lee SY, Kim CW, Kang JH, Seo NK. Unmet healthcare needs depending on employment status. *Health Policy* 2015; **119**(7): 899-906.

68. Levesque JF, Pineault R, Hamel M, et al. Emerging organisational models of primary healthcare and unmet needs for care: insights from a population-based survey in Quebec province. *BMC Fam Pract* 2012; **13**: 66.

69. Li X, Chen M, Wang Z, Si L. Forgone care among middle aged and elderly with chronic diseases in China: evidence from the China Health and Retirement Longitudinal Study Baseline Survey. *BMJ Open* 2018; **8**(3): e019901.

70. Lindstrom C, Rosvall M, Lindstrom M. Socioeconomic status, social capital and self-reported unmet health care needs: A population-based study. *Scandinavian Journal of Public Health* 2017; **45**(3): 212-21.

71. Lindström C, Rosvall M, Lindström M. Differences in unmet healthcare needs between public and private primary care providers: A population-based study. *Scand J Public Health* 2018; **46**(4): 488-94.

72. Listl S. Cost-related dental non-attendance in older adulthood: evidence from eleven European countries and Israel. *Gerodontology* 2016; **33**(2): 253-9.

73. Maille G, Saliba-Serre B, Ferrandez AM, Ruquet M. Use of care and the oral health status of people aged 60 years and older in France: results from the National Health and Disability Survey. *Clin Interv Aging* 2017; **12**: 1159-66.

74. Marcin SP, Butler JR, Connelly LB. Unmet medical needs and health care accessibility in seven countries of Eastern Europe. (<https://mpra.ub.uni-muenchen.de/id/eprint/75619>). 2014.

75. Marshall EG. Do young adults have unmet healthcare needs? *J Adolesc Health* 2011; **49**(5): 490-7.

76. McLeod KE, Karim ME. The relationship between mood disorder diagnosis and experiencing an unmet health-care need in Canada: findings from the 2014 Canadian Community Health Survey. *Journal of Mental Health* 2020: 1-13.

77. Mielck A, Kiess R, Knesebeck OVD, Stirbu I, Kunst AE. Association between forgone care and household income among the elderly in five Western European countries analyses based on survey data from the SHARE-study. *BMC Health Services Research* 2009; **9**.

78. Newacheck PW, Hughes DC, Hung Y, Wong S, Stoddard JJ. The unmet health needs of America's children. *Pediatrics* 2000; **105**(4): 989-97.

79. Njagi P, Arsenijevic J, Groot W. Cost-related unmet need for healthcare services in Kenya. *BMC health services research* 2020; **20**(1): 322.

80. Pathman DE, Fowler-Brown A, Corbie-Smith G. Differences in access to outpatient medical care for black and white adults in the rural South. *Med Care* 2006; **44**(5): 429-38.

81. Peterson LE, Litaker DG. County‐level poverty is equally associated with unmet health care needs in rural and urban settings. *The Journal of Rural Health* 2010; **26**(4): 373-82.

82. Petrelli A, Rosano A, Rossi A, Mirisola C, Cislaghi C. The geography and economics of forgoing medical examinations or therapeutic treatments in Italy during the economic crisis. *BMC Public Health* 2019; **19**(1): 1202.

83. Popovic N, Terzic-Supic Z, Simic S, Mladenovic B. Predictors of unmet health care needs in Serbia; Analysis based on EU-SILC data. *PLoS One* 2017; **12**(11): e0187866.

84. Prazeres F, Santiago L. Relationship between health-related quality of life, perceived family support and unmet health needs in adult patients with multimorbidity attending primary care in Portugal: a multicentre cross-sectional study. *Health and quality of life outcomes* 2016; **14**(1): 1-11.

85. Reichard A, Stransky M, Phillips K, McClain M, Drum C. Prevalence and reasons for delaying and foregoing necessary care by the presence and type of disability among working-age adults. *Disability and Health Journal* 2017; **10**(1): 39-47.

86. Rew L, Resnick M, Beuhring T. Usual sources, patterns of utilization, and foregone health care among Hispanic adolescents. *Journal of Adolescent Health* 1999; **25**(6): 407-13.

87. Rhee TG, Marottoli RA, Van Ness PH, Levy BR. Impact of Perceived Racism on Healthcare Access Among Older Minority Adults. *American Journal of Preventive Medicine* 2019; **56**(4): 580-5.

88. Rivenbark JG, Ichou M. Discrimination in healthcare as a barrier to care: experiences of socially disadvantaged populations in France from a nationally representative survey. *BMC Public Health* 2020; **20**(1): 31.

89. Ro YS, Shin SD, Jeong J, et al. Evaluation of demands, usage and unmet needs for emergency care in Yaoundé, Cameroon: A cross-sectional study. *BMJ Open* 2017; **7**(2).

90. Ronksley PE, Sanmartin C, Quan H, et al. Association between chronic conditions and perceived unmet health care needs. *Open Med* 2012; **6**(2): e48-58.

91. Samargia LA, Saewyc EM, Elliott BA. Foregone mental health care and self-reported access barriers among adolescents. *J Sch Nurs* 2006; **22**(1): 17-24.

92. Sanmartin C, Houle C, Tremblay S, Berthelot JM. Changes in unmet health care needs. *Health Rep* 2002; **13**(3): 15-21.

93. Schoen C, Osborn R, Squires D, Doty MM. Access, affordability, and insurance complexity are often worse in the United States compared to ten other countries. *Health Affairs* 2013; **32**(12): 2205-15.

94. Seung Eun L, Miyeon Y, Chul-Woung K, Tae-Ho Y, Dongjin K, Jihee C. Neighborhood Deprivation and Unmet Health Care Needs: A Multilevel Analysis of Older Individuals in South Korea. *Osong Public Health & Research Perspectives* 2019; **10**(5): 295-306.

95. Shi L, Lebrun LA, Tsai J. The influence of English proficiency on access to care. *Ethnicity and Health* 2009; **14**(6): 625-42.

96. Shi L, Stevens GD. Vulnerability and unmet health care needs: The influence of multiple risk factors. *Journal of General Internal Medicine* 2005; **20**(2): 148-54.

97. Silver EJ, Stein RE. Access to care, unmet health needs, and poverty status among children with and without chronic conditions. *Ambulatory pediatrics : the official journal of the Ambulatory Pediatric Association* 2001; **1**(6): 314-20.

98. Smaldone A, Honig J, Byrne MW. Delayed and forgone care for children with special health care needs in New York State. *Matern Child Health J* 2005; **9**(2 Suppl): S75-86.

99. Stein J, Liegert P, Dorow M, König H-H, Riedel-Heller SG. Unmet health care needs in old age and their association with depression–results of a population-representative survey. *J Affect Disord* 2019; **245**: 998-1006.

100. Stransky ML. Unmet Needs for Care and Medications, Cost as a Reason for Unmet Needs, and Unmet Needs as a Big Problem, due to Health-Care Provider (Dis)Continuity. *J Patient Exp* 2018; **5**(4): 258-66.

101. Tambor M, Pavlova M, Rechel B, Golinowska S, Sowada C, Groot W. The inability to pay for health services in Central and Eastern Europe: evidence from six countries. *Eur J Public Health* 2014; **24**(3): 378-85.

102. Thammatacharee N, Tisayaticom K, Suphanchaimat R, et al. Prevalence and profiles of unmet healthcare need in Thailand. *BMC Public Health* 2012; **12**: 923.

103. Towne SD, Jr. Socioeconomic, Geospatial, and Geopolitical Disparities in Access to Health Care in the US 2011-2015. *Int J Environ Res Public Health* 2017; **14**(6).

104. Travers JL, Cohen CC, Dick AW, Stone PW. The great American Recession and forgone healthcare: Do widened disparities between African-Americans and Whites remain? *PLoS ONE* 2017; **12**(12).

105. Warfield ME, Gulley S. Unmet need and problems accessing specialty medical and related services among children with special health care needs. *Maternal and child health journal* 2006; **10**(2): 201-16.

106. Washington DL, Bean-Mayberry B, Riopelle D, Yano EM. Access to care for women veterans: delayed healthcare and unmet need. *J Gen Intern Med* 2011; **26**: 655-61.

107. Weathers A, Minkovitz C, O'Campo P, Diener-West M. Access to care for children of migratory agricultural workers: factors associated with unmet need for medical care. *Pediatrics* 2004; **113**(4): e276-82.

108. Wiltshire JC, Person SD, Kiefe CI, Allison JJ. Disentangling the influence of socioeconomic status on differences between African American and white women in unmet medical needs. *Am J Public Health* 2009; **99**(9): 1659-65.

109. Winkelman TNA, Segel JE, Davis MM. Medicaid enrollment among previously uninsured Americans and associated outcomes by race/ethnicity-United States, 2008-2014. *Health Serv Res* 2019; **54**: 297-306.

110. Wisk LE, Gangnon R, Vanness DJ, Galbraith AA, Mullahy J, Witt WP. Development of a novel, objective measure of health care-related financial burden for U.S. families with children. *Health Serv Res* 2014; **49**(6): 1852-74.

111. Wu Z, Penning MJ, Schimmele CM. Immigrant status and unmet health care needs. *Can J Public Health* 2005; **96**(5): 369-73.

112. Yamada T, Chen CC, Murata C, et al. Access disparity and health inequality of the elderly: unmet needs and delayed healthcare. *Int J Environ Res Public Health* 2015; **12**(2): 1745-72.

113. Yu JW, Adams SH, Burns J, Brindis CD, Irwin CE, Jr. Use of mental health counseling as adolescents become young adults. *J Adolesc Health* 2008; **43**(3): 268-76.

114. Stella MY, Singh GK. Household language use and health care access, unmet need, and family impact among CSHCN. *Pediatrics* 2009; **124**(Supplement 4): S414-S9.

115. Zhu. Unmet needs in long-term care and their associated factors among the oldest old in China. *BMC Geriatr* 2015; **15**: 46.

116. Zhu Y, Osterle A. Rural-urban disparities in unmet long-term care needs in China: The role of the hukou status. *Soc Sci Med* 2017; **191**: 30-7.

117. Zimmer-Gembeck MJ, Alexander T, Nystrom RJ. Adolescents report their need for and use of health care services. *J Adolesc Health* 1997; **21**(6): 388-99.

118. K A, Z H, L S. Likelihood that expectations of informal care will be met at onset of caregiving need: a retrospective study of older adults in the USA. *BMJ open* 2017; **7**(12): e017791.

119. Andrade TB, FB A. Unmet need for assistance with activities of daily life among older adults in Brazil. *Rev Saude Publica* 2018; **52**: 75.

120. Fu Y, Guo Y, Bai X, Chui E. Factors associated with older people's long-term care needs: a case study adopting the expanded version of the Anderson Model in China. *MC Geriatr* 2017; **17**(1): 38.

121. Hass Z, DePalma G, Craig BA, Xu H, LP S. Unmet Need for Help With Activities of Daily Living Disabilities and Emergency Department Admissions Among Older Medicare Recipients. *Gerontologist* 2017; **57**(2): 206-10.

122. He S, Craig BA, Xu H, et al. Unmet Need for ADL Assistance Is Associated With Mortality Among Older Adults With Mild Disability. *Ser A Biol Sci Med Sci* 2015; **70**(9): 1128-32.

123. V H, A S, B M. Determinants of Unmet Needs among Slovenian Old Population. *Zdravstveno varstvo* 2016; **55**(1): 78-85.

124. Hu B, Wang J. Unmet long-term care needs and depression: The double disadvantage of community-dwelling older people in rural China. *Health Soc Care Community* 2019; **27**(1): 126-38.

125. H L. Involvement of informal and formal service providers: meeting the home care needs of older adults with severe functional impairments. *Home health care services quarterly* 2006; **25**(3): 167-83.

126. Tennstedt S, McKinlay J, L K. Unmet need among disabled elders: a problem in access to community long term care? *Soc Sci Med* 1994; **38**(7): 915-24.

127. X X, R A, A H. Depression and Unmet Needs for Assistance With Daily Activities Among Community-Dwelling Older Adults. *Gerontologist* 2018; **58**(3): 428-37.

128. Zhen Z, Feng Q, Gu D. The impacts of unmet needs for long-term care on mortality among older adults in China. *Journal of Disability Policy Studies* 2015; **25**(4): 243-51.

129. A Z, X X. Anxiety and Unmet Needs for Assistance With Daily Activities Among Older Adults. *Journal of aging and health* 2020; **32**(5): 491-500.
